# Tumour deposits are associated with worse survival than extranodal extension; a network meta‐analysis on tumour nodules in colorectal cancer

**DOI:** 10.1111/his.15301

**Published:** 2024-08-28

**Authors:** Nelleke P M Brouwer, Shannon van Vliet, Joanna IntHout, Johannes H W De Wilt, Femke Simmer, Niek Hugen, Iris D Nagtegaal

**Affiliations:** ^1^ Department of Pathology Radboud University Medical Center Nijmegen the Netherlands; ^2^ Department for Health Evidence Radboud University Medical Center Nijmegen the Netherlands; ^3^ Department of Surgery Radboud University Medical Center Nijmegen the Netherlands; ^4^ Department of Surgery Netherlands Cancer Institute Amsterdam the Netherlands; ^5^ Department of Surgery Rijnstate Hospital Arnhem the Netherlands

**Keywords:** colorectal cancer, extranodal extension, lymph node metastases, network meta‐analysis, prognostic value, staging, tumour deposits

## Abstract

Lymph node metastases (LNM) play a central role in the tumour–node–metastasis (TNM) classification for colorectal cancer (CRC), with extranodal extension (ENE) as an adverse feature. ENE has never been directly compared to tumour deposits (TD). The aim of this study was to perform an up‐to‐date systematic review, including a network meta‐analysis to compare their prognostic value. A comprehensive search was conducted on PubMed, Embase, Web of Science and Cochrane databases to identify all prognostic studies on ENE and TD. A total of 20 studies were included, with 7719 cases. The primary outcome was 5‐year disease‐free survival (DFS); secondary outcomes were overall survival (OS) and disease‐specific survival (DSS). Frequentist paired and network meta‐analyses were performed using the *netmeta* package in R. For univariable DFS analysis, LNM + TD+ cases had a significantly worse outcome compared with LNM + ENE+ cases [hazard ratio (HR) = 1.27, 95% confidence interval (CI) = 1.06–1.53], which was no longer significant for multivariable DFS analysis (HR = 1.13, 95% CI = 0.87–1.46). All OS and multivariable DSS analyses showed a significantly worse outcome for LNM + TD+ cases compared with LNM + ENE cases. For all outcomes, both LNM + TD+ and LNM + ENE+ had a significantly increased hazard compared with LNM+ cases. This study shows that there is a trend towards worse outcome for LNM + TD+ than LNM + ENE+, not statistically significant in multivariable DFS analysis. Both groups perform significantly worse than cases with LNM only. To improve the accuracy of CRC staging, we recommend to put more emphasis on both ENE and TD in the TNM classification, with the most prominent role for TD.

## Graphical abstract

This systematic review compares LNM+, LNM + TD+ and LNM + ENE+ cases using network meta‐analyses. As LNM+TD+ has a worse outcome than LNM +E NE+ and both groups perform worse than cases with LNM only, more emphasis on both ENE and TD in the TNM classification is needed, with the most prominent role for TD.

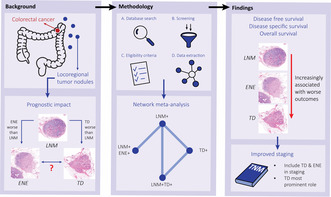

**Abbreviations**
CRC: colorectal cancer
DFS: disease free survival
DSS: disease specific survival
ENE: extranodal extension
HR: hazard ratio
LNM: lymph node metastasis
LNM+: cases with only lymph node metastases
LNM+ENE+: cases with lymph node metastases and extranodalextension
LNM+TD+: cases with lymph node metastases and tumour deposits
OS: overall survival
TD: tumour deposit
TD+: cases with only tumour deposits
TNM: tumour node metastasis

## Introduction

Colorectal cancer (CRC) staging according to the tumour–node–metastasis (TNM) system aids clinicians in prognostic stratification and treatment decisions. Currently, lymph node metastases (LNM) are a central prognostic factor in staging, determining the need for adjuvant treatment.^1^ However, other histological factors, including tumour deposits (TD) and extranodal extension of LNM (ENE), have also shown significant prognostic value.^2^, ^3^, ^4^

TD are defined as isolated tumour cell nodules in the mesocolonic fat without evidence of lymph node tissue, vascular and neural structures.^5^ Since first described by Gabriel *et al*. in 1935,^6^ the prognostic value of TD has been widely established.^2^, ^3^ However, TD were only defined as a separate entity in the 7th edition of the TNM with the introduction of the N1c category, deeming them only clinically relevant in the absence of LNM.^7^ In case of ENE, tumour cells penetrate the nodal capsule and extend into the perinodal tissue. The current TNM classification for CRC does not incorporate the presence of ENE, despite ample evidence of worse survival.^4^, ^8^

It has been suggested that ENE is a necessary step in the progression from LNM towards TD.^5^, ^9^ Previous studies show the additional prognostic value of both ENE and TD compared with LNM only,^3^, ^4^ but it is unclear how the prognostic value of ENE and TD compares. Both are indicative of more aggressive tumour biology. With TD as a possible progression of ENE, they are expected to have more prognostic effect than ENE, but evidence for this hypothesis is lacking.

A more comprehensive understanding of the prognostic value of LNM, ENE and TD in CRC is needed to improve the accuracy of the TNM classification. The lack of a study comparing LNM, ENE and TD could be overcome by a network meta‐analysis, which allows for inclusion of multiple studies on different comparisons in a single analysis.^10^ This study aims to analyse the prognostic value of LNM, ENE and TD in a paired meta‐analysis as well as to perform a frequentist network meta‐analysis to compare LNM, ENE and TD in a single analysis.

## Methods

All analyses in this study were performed according to the Preferred Reporting Items for Systematic reviews and Meta‐Analyses (PRISMA) extension statement for network meta‐analysis.^11^ The study protocol was registered in the Prospective Register of Systematic Reviews (PROSPERO‐ID: CRD42023470433). The compared groups were cases with only LNM (LNM+), cases with LNM and ENE (LNM + ENE+), cases with TD but no LNM (TD+) and cases with LNM and TD (LNM + TD+).

### Literature search, eligibility criteria and data extraction

A comprehensive systematic literature search for published studies was performed on 19 October 2023 by using the PubMed, Embase, Web of Science and Cochrane databases. The detailed search strategy is shown in Supporting information, Table S1. Two additional publications were added by manual cross‐referencing.

Only original studies published in English with at least 100 patients were selected. In case of overlapping patient data, results of the largest study were included in this meta‐analysis. Studies in which histology was not reviewed were excluded, as it is known that the definition of TD is complex and high interobserver variation exists.^12^ Lack of reassessment of the tumour nodules by an expert pathologist can therefore lead to inaccuracy and bias. Also, those that included only neoadjuvant treated cases were excluded. If studies reported different cohorts based on tumour location (colon or rectum), nodal stage (N1 or N2), or if they included a test and validation cohort, these were separately analysed. Screening of publications was performed independently by two investigators (N.P.M.B. and S.V.) and disagreements were resolved by a third (I.D.N.).

Baseline characteristics and data on 5‐year disease‐free survival (DFS, the time from diagnosis to recurrence or death), overall survival (OS, the time from diagnosis to death) and disease‐specific survival (DSS, the time from diagnosis to death caused by the cancer) were extracted from all studies. Data from both univariable (i.e. only including the presence of tumour nodules, without consideration of other characteristics) and multivariable (i.e. also including other clinicopathological characteristics to correct for their effect) analyses were extracted. If no hazard ratio (HR) was reported, it was extracted from the Kaplan–Meier curves using Parmar estimation.^13^

### Quality assessment

The methodological quality of enrolled studies was assessed by two investigators (N.P.M.B. and S.V.) independently and disagreements were resolved by a third (I.D.N.). Study quality was assessed using a scale designed for the evaluation of histopathological studies.^14^, ^15^

### Statistical analyses

A conventional random‐effects meta‐analysis was used to obtain direct estimates between LNM+, TD+, LNM + ENE+ and LNM + TD+ if two or more studies were available, using HR as the effect estimate. The inverse variance method was used, using a restricted maximum‐likelihood estimator for *τ* ^2^ as well as Hartung–Knapp adjustment. The statistical heterogeneity between prognostic effects across studies was assessed using the *I* ^2^ statistics.^16^

Paired meta‐analyses are limited to two groups only. Therefore, a frequentist network meta‐analysis was conducted to simultaneously compare the effects of more than two groups. Due to the inherent between‐study heterogeneity, a random‐effects network meta‐analysis was performed to estimate the effects for each pairwise comparison in the network with their 95% confidence intervals (95% CI), using a restricted maximum‐likelihood estimator for *τ* ^2^. By combining direct estimates with indirect estimates, a network meta‐analysis yields a mixed effect estimate. An estimate for the indirect HR of LNM + TD+ versus LNM + ENE+ cases can be obtained through the direct comparison of LNM + TD+ versus LNM+ cases and LNM + ENE+ versus LNM+ cases, by calculating the difference in the natural logarithms of the HRs between the two direct comparisons and exponentiating this difference. A network plot was generated to visually display the comparative relationships among the different tumour nodules for the different outcomes. Forest plots showing the direct and indirect results of the network meta‐analysis were generated to assess inconsistency and the *P*‐score was analysed to rank the different types of tumour nodules based on their prognostic value.^17^ The *P*‐score is obtained by estimating the effect sizes of pairwise comparisons and ranks the compared groups with a value between 0 and 1, where a higher *P*‐score means a stronger association with worse survival. Funnel plots were generated to qualitatively evaluate publication bias.

The paired meta‐analyses were performed using the *meta* package, and the network meta‐analyses using the *netmeta* package, both of R software (version 4.3.1).^18^, ^19^, ^20^

## Results

### Study selection, quality assessment and publication bias

**Figure 1:**
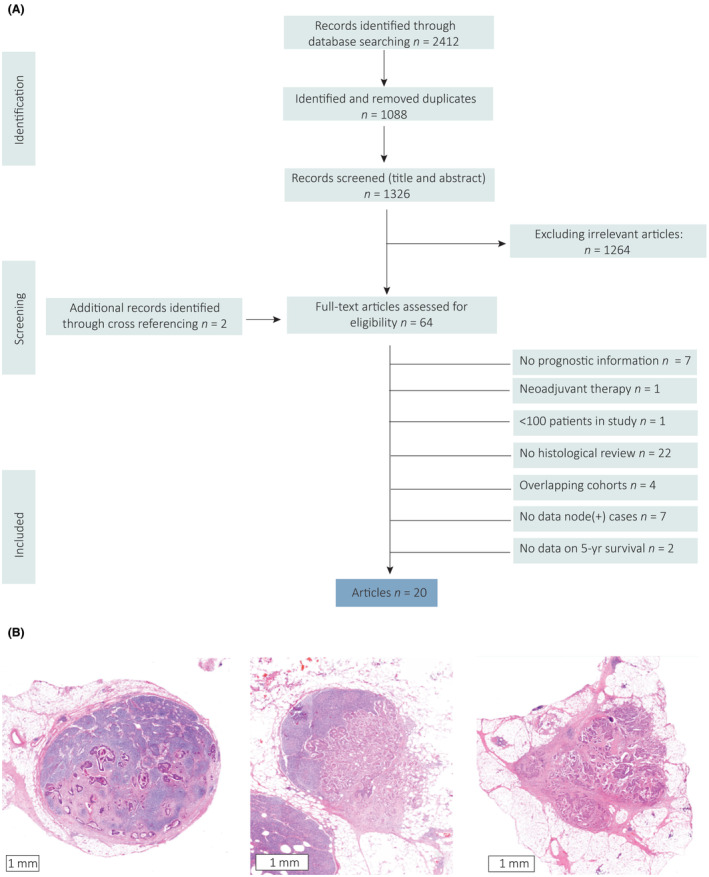
**A**, Preferred Reporting Items for Systematic reviews and Meta‐Analyses (PRISMA) flow chart showing selection of articles for review. **B**, Haematoxylin and eosin‐stained images showing the histology from left to right of a lymph node metastasis (LNM), extranodal extension (ENE) and tumour deposit (TD). LNM were defined as tumour cells inside a lymph node in the pericolorectal fat. ENE was defined as the extension of tumour cells through the lymph node capsule. TD were defined as clusters of tumour cells in the pericolorectal fat without recognisable remnants of a lymph node, vascular invasion or perineural invasion, following the definitions in the tumour–node–metastasis (TNM)8.^1^ [Color figure can be viewed at wileyonlinelibrary.com]

A total of 2412 studies were retrieved by the literature search (Figure 1A) and after exclusion of duplicates, title and abstract screening and full article assessment, 20 studies were included (Tables 1 and 2). Examples of the different tumour nodules included in the analyses are shown in Figure 1B. All studies were subjected to quality assessment based on the reporting of predefined items important for histological studies (Supporting information, Table S2). The percentage of reported items ranged from 53 to 87%.

**Table 1:** Baseline characteristics of included studies in the meta‐analysis of extranodal extension

Author	Year	Country	Rectal cancer cases	Inclusion period	Stages	Selected stages	Total no. of cases	No. selected cases for analyses	No. cases ENE+ (%)		Meta‐analysis outcome univariable	Meta‐analysis outcome multivariable
Al Sahaf *et al*.^32^	2011	Ireland	0%	NR	III	III	194	114	78	(68%)	DFS, DSS	DFS, DSS
Ambe *et al*.^33^	2018	Germany	31%	2009–2013	III/IV (N+)	III/IV (N+)	147	147	78	(53%)	OS	–
Brabender *et al*.^34^	2012	Germany	100%	1997–2007	I–IV	III/IV (N+)	231	95	52	(55%)	OS	–
Kim *et al*.^35^	2016	South Korea	43%	2005–2010	III/IV	III/IV (N+)	419	230	108	(47%)	DFS, OS	DFS, OS
Kim *et al*., |colon^36^	2019	South Korea	0%	2003–2011	III	III	1363	1363	551	(40%)	DF	DFS
Kim *et al*., |rectum^36^	2019	South Korea	100%	2003–2011	III	III	983	983	479	(49%)	DFS	DFS
Komori *et al*.^37^	2013	Japan	100%	1979–2001	III	III	101	52	19	(37%)	DFS, OS	DFS, OS
Li *et al*.^38^	2021	China	69%	2014–2018	III/IV (N+)	III/IV (N+)	389	389	244	(63%)	OS	OS
Puppa *et al*.^39^	2007	Italy	0%	1988–1999	III/IV (N+)	III/IV (N+)	228	228	99	(43%)	DFS, DSS	DFS, DSS
Ueno *et al*.^40^	1998	Japan	100%	1981–1993	I–IV	III/IV (N+)	218	93	35	(38%)	OS	–
Wind *et al*.^41^	2008	Netherlands	0%	1994–2005	III	III	111	111	58	(53%)	DFS	DFS

DFS, disease‐free survival; DSS, disease‐specific survival; NR, not reported; OS, overall survival.

**Table 2:** Baseline characteristics of included studies in the meta‐analysis of tumour deposits

Author	Year	Country	Rectal cancer cases	Inclusion period	Selected stages	Stages	Total no. of cases	No. of selected cases for analyses	No. of cases TD+ (%)		Meta‐analysis outcome univariable	Meta‐analysis outcome multivariable
Al Sahaf *et al*.^32^	2011	Ireland	0%	NR	III	III	194	114	33	(29%)	DFS, DSS	DSS
Belt *et al*.^42^	2010	Netherlands	23%	1996–2005	III	II/III	870	212	43	(20%)	DFS	–
Goldstein *et al*.^43^	2000	USA	0%	1973–1984/ 1986–1992	III	III	400	400	89	(22%)	–	DFS
Kim *et al*.^35^	2016	South Korea	43%	2005–2010	III/IV (N+)	III/IV	419	230	72	(31%)	DFS, OS	DFS, OS
Landau *et al*.^44^	2019	USA	0%	2011–2013	III	II/III	256	136	48	(35%)	DFS	DFS
Nagayoshi *et al*.^45^	2014	Japan	22%	1999–2006	III	II/III	344	172	27	(16%)	–	DFS, OS
Puppa *et al*.^39^	2007	Italy	0%	1988–1999	III/IV (N+)	III/IV (N+)	228	228	100	(44%)	DFS, DSS	–
Qiu *et al*.^46^	2011	China	50%	2000–2005	III	III	1215	936	167	(14%)	DFS	DFS
Shimada *et al*.^47^	2010	Japan	100%	2000–2005	III (N+)	I–III	214	81	59	(73%)	DFS	–
Tateishi *et al*. Colon^48^	2001	Japan	44%	1985–1995	III/IV (N+)	I–IV	544	97	19	(20%)	DSS	–
Tateishi *et al*. |Rectum^48^	2001	Japan	44%	1985–1995	III/IV (N+)	I–IV	544	149	78	(52%)	DSS	–
Ueno *et al*.^49^	2014	Japan	31%	1994–1998	III	II/III	1716	315	83	(23%)	DFS	–
Winterfeld *et al*.^50^	2014	Germany	39%	2003–2007	III	I–IV	414	134	63	(47%)	–	DFS, OS, DSS
Yabata *et al*.^51^	2014	Japan	20%	2000–2008	III	I–III	464	170	43	(25%)	OS	OS

DFS, disease‐free survival; DSS, disease‐specific survival; NR, not reported; OS, overall survival.

For all network meta‐analyses, the direct and indirect estimates were calculated, and no indication of inconsistency was detected (Supporting information, Figure S1). Publication bias was assessed by generating network funnel plots based on the different survival outcomes, which did not reveal marked asymmetry among the studies, indicating the absence of significant publication bias (Supporting information, Figure S2). Sensitivity analyses were performed for subgroups based on tumour location and geographical origin of the patients but this had no impact on outcome.

### Disease‐free survival

**Figure 2:**
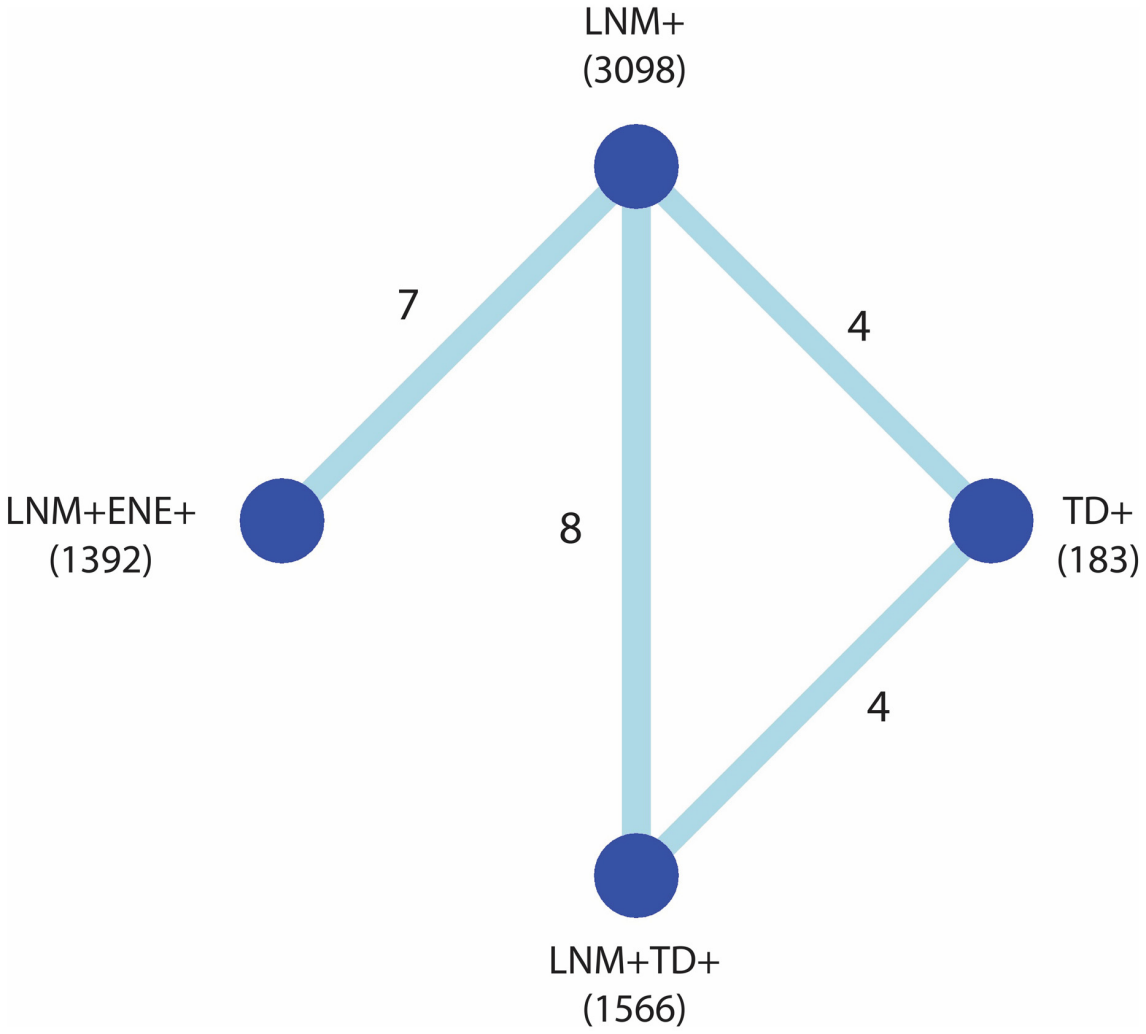
Network comparisons for the frequentist network meta‐analysis for disease‐free survival (univariable). The nodes in the network represent the groups that are compared, including the number of patients for these groups. The arms show the comparisons for which direct evidence is available, the numbers indicating the number of studies including this comparison. This direct evidence is shown in detail in the conventional paired meta‐analyses in Figure 3, with the comparison LNM + TD+ versus LNM+ in Figure 3**A**, LNM + ENE+ versus LNM+ in Figure 3**B**, LNM+ versus TD+ in Figure 3**C** and LNM + TD+ versus TD+ in Figure 3**D**. [Color figure can be viewed at wileyonlinelibrary.com]

**Figure 3:**
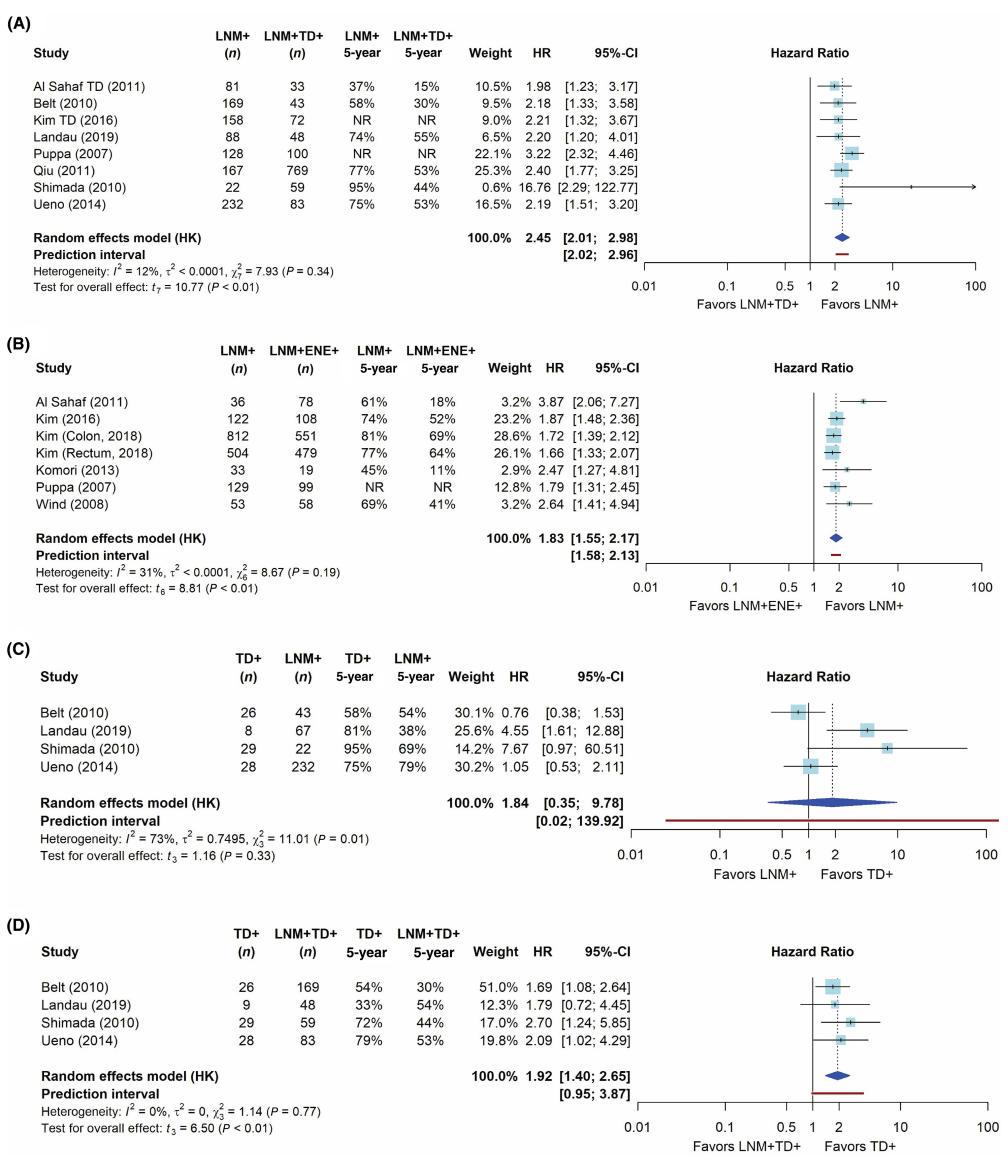
Forest plots showing the direct pairwise comparisons of disease‐free survival (univariable). **A**, Pairwise comparison between LNM + TD+ and LNM+ cases. **B**, Pairwise comparison between LNM + ENE+ and LNM+ cases. **C**, Pairwise comparison between LNM+ and TD+ cases. **D**, Pairwise comparison between LNM + TD+ and TD+ cases. [Color figure can be viewed at wileyonlinelibrary.com]

The network of comparisons for the frequentist meta‐analysis of univariable DFS is shown in Figure 2; networks for the other outcome measures are provided in Supporting information, Figure S3. The network meta‐analysis is based on the direct evidence from the paired analyses. In these analyses, both LNM + TD+ and LNM + ENE+ cases had a significantly worse DFS compared with LNM+ cases with a HR of 2.45 (95% CI = 2.01–2.98, Figure 3A) and 1.83 (95% CI = 1.55–2.17, Figure 3B), respectively. The network meta‐analysis yielded a significantly worse DFS for LNM + TD+ compared with LNM + ENE+ cases (HR = 1.28, 95% CI = 1.07–1.54, Table 3). When ranking tumour nodules by prognostic value based on the *P*‐score, LNM + TD+ had the largest impact on DFS (Supporting information, Figure S4).

**Table 3:** Mixed effect hazard ratio estimates from the frequentist network meta‐analyses for the three univariable survival outcomes

Outcomes	Number of cohorts (patients)	Comparison	LNM+	TD+	LNM + ENE+
Disease‐free survival	LNM+: 19 (3098)				
	TD+: 8 (183)	TD+	1.04 [0.79–1.37]	–	–
	LNM + ENE+: 7 (1392)	LNM + ENE+	1.83 [1.64–2.05]	1.77 [1.31–2.38]	–
	LNM + TD+: 12 (1566)	LNM + TD+	2.35 [2.03–2.73]	2.27 [1.74–2.95]	1.28 [1.07–1.54]
Disease‐specific survival	LNM+: 8 (656)				
	TD+: 4 (69)	TD+	1.35 [0.72–2.54]	–	–
	LNM + ENE+: 2 (177)	LNM + ENE+	2.16 [1.10–4.23]	1.60 [0.64–4.02]	–
	LNM + TD+: 6 (275)	LNM + TD+	1.96 [1.24–3.09]	1.45 [0.79–2.66]	0.91 [0.40–2.04]
Overall survival	LNM+: 8 (755)				
	TD+: 0 (0)	TD+	–	–	–
	LNM + ENE+: 6 (536)	LNM + ENE+	2.18 [1.74–2.72]	–	–
	LNM + TD+: 2 (116)	LNM + TD+	3.97 [2.83–5.59]	–	1.83 [1.22–2.74]

Statistically significant hazard ratios are coloured blue and non‐significant hazard ratios are coloured orange.

For the analysis on multivariable DFS, paired analyses show that both LNM + ENE+ and LNM + TD+ cases had significantly worse DFS compared to LNM+ cases with HR of 1.74 (95% CI = 1.22–2.49) and 1.50 (95% CI = 1.25–1.82; Supporting information, Figure S5A). The network analysis resulted in a DFS for LNM + TD+ cases compared to LNM + ENE+ cases, which was not significantly different (HR = 1.13; 95% CI = 0.87–1.46, Supporting information, Table S3).

### Disease‐specific survival

For univariable DSS, the pairwise analyses showed a worse DSS for LNM + ENE+ and LNM + TD+ cases when compared to LNM+ cases (Figure S5B). High heterogeneity based on the *I* ^2^ statistic was found for the comparison between LNM + ENE+ and LNM+ cases. The network meta‐analysis showed that DSS was not statistically different between for LNM + TD+ cases compared with LNM + ENE+ cases (Table 3).

The multivariable DSS results were in line with the results for DFS and OS, as the network analysis showed a significantly worse DSS for LNM + TD+ cases compared with LNM + ENE+ cases (HR = 1.82; 95% CI = 1.08–3.08, Supporting information, Table S3).

### Overall survival

For univariable OS, the pairwise comparisons yielded worse OS for LNM + ENE+ and LNM + TD+ cases compared with LNM+ cases with a HR of 3.97 (95% CI = 2.07–7.65) and 2.21 (95% CI = 1.49–3.28), respectively (Figure S5B). The network analysis showed a significantly worse OS for LNM + TD+ compared with LNM + ENE+ (HR = 1.83; 95% CI = 1.22–2.74, Table 2).

For pairwise comparisons of multivariable OS, a worse OS was found for LNM + ENE+ and LNM + TD+ cases compared with LNM+ cases with a HR of 2.56 (95% CI = 1.98–3.31) and 1.58 (95% CI = 1.07–2.34), respectively (Figure S5C). The network meta‐analysis of the multivariable OS data showed a significantly worse OS for LNM + TD+ compared with LNM + ENE+ cases (HR = 1.62; 95% CI = 1.07–2.44; Supporting information, Table S3).

## Discussion

Although the presence of both ENE and TD have a strong association with worse survival, they have not been compared or analysed together previously.^3^, ^4^ We confirmed that the presence of both TD and ENE leads to worse survival in node‐positive CRC. Furthermore, using a network meta‐analysis, we also showed that there is a trend towards worse survival for LNM + TD+ cases compared with LNM + ENE+ cases, illustrated by a HR of 1.27 (95 CI = 1.06–1.53) and 1.83 (95% CI = 1.22–2.74) for univariable 5‐year DFS and OS, respectively, although this difference was not statistically significant in the multivariable meta‐analysis for DFS.

The results from this study are in line with previous systematic reviews that performed paired meta‐analyses showing a worse prognosis for TD and ENE‐positive cases.^2^, ^3^, ^4^, ^8^, ^21^ The current study adds to this by including the most recent publications on ENE as well as by indirectly comparing LNM + ENE+ and LNM + TD+ cases. Furthermore, several of the meta‐analyses on TD included studies in which the tumour nodules were not histologically revised (reassessing all nodules by an expert pathologist to distinguish between ENE and TD).^2^, ^21^ Histological revision is crucial to differentiate between ENE and TD, as it is known that the definition of TD is complex, and the lack of reassessment by an expert can lead to biased results.^12^ Therefore, revision by an expert pathologist was an inclusion criterion in the current study.

The only study that included data comparing LNM + TD+ with LNM + ENE+ cases was small and did not have this comparison as the main objective. Both groups had significantly worse DFS compared with LNM only, but between LNM + TD+ and LNM + ENE+ cases no significant difference in DFS was found, possibly due to insufficient power.^22^

The increasingly worse outcome from LNM to ENE and TD could be explained by more aggressive biology and thereby the increased complexity of the shape of these nodules. When tumour cells in LNM grow through the lymphatic capsule and develop ENE, they have acquired characteristics enabling migration through the extracellular matrix and across histological borders, relevant for distant metastasis formation.^23^ Similarly, TD are characterised by the destruction of histological boundaries. We have previously shown by transcriptome profiling that TD are indeed characterised by a more invasive phenotype (including epithelial mesenchymal transition, invasion and matrix remodelling) compared with LNM.^24^ This destruction of histological borders causes increasingly complex shapes of tumour nodules with less rounded shapes and more protrusions. Indeed, the complexity of the shape of tumour nodules increases from LNM to ENE to TD. Furthermore, increasingly complex shapes were found to be continuously associated with worse DFS.^25^ This finding is confirmed by the current study that ranked LNM + TD+ cases as having the worse outcomes, followed by LNM + ENE+ cases and cases with only LNM (Supporting information, Figure S4). However, further research is needed to investigate if this invasive biology, reflected in the more complex shape of ENE/TD, allows the tumour cells in these nodules to directly spread to other organs and form distant metastases, or whether these are merely biomarkers.

The use of a network meta‐analysis for histopathological parameters is novel. It enabled us to compare the prognostic value of LNM+, TD+, LNM + ENE+ and LNM + TD+ cases in one single analysis, generating a uniquely comprehensive overview of the prognostic value of different types of tumour nodules in CRC, which was previously limited to paired comparisons. It is important to take some caution when interpreting the results in this innovative approach. Conventionally, network meta‐analyses are applied to randomised clinical trials, which are different from the cohorts such as those used in the current study, the most important difference being the more uncontrolled nature of the reference group in histopathological studies. In the current study, LNM+ cases were used as the reference group to link the outcomes of LNM + ENE+ and LNM + TD+ cases. However, the studies that compared LNM + ENE+ with LNM+ cases were uncontrolled for TD prevalence. Therefore, a bias due to higher prevalence of TD in the LNM + ENE+ group is possible, which would enhance its HR. Similarly, the presence of ENE could have biased the results for the LNM + TD+ group, although if these biases are real, then this would mean that the true difference is larger than we estimated. High heterogeneity was found, mainly for DSS, which can be explained by the low number of cases in the included studies, as DSS is an infrequently reported outcome measure.^26^ Also, it is not possible to compare the results from the network meta‐analysis for the comparison of LNM + ENE+ and LNM + TD with a paired meta‐analysis, due to the lack of direct evidence for this comparison in the literature. However, the comparisons from the network meta‐analyses and their paired meta‐analyses in this study were generally in line with each other. Lastly, there is currently no evidence in the literature for the prognostic impact of LNM + ENE+TD+ cases, making it impossible to determine whether the prognostic effects of both ENE and TD are additive. Future research should address this.

Currently, the presence of ENE is not relevant for the final TNM stage of CRC cases and TD are included in the N1c category in the TNM, but only in the absence of LNM, despite their impact on prognosis.^1^ The results from the current meta‐analyses show that both ENE and TD are associated with worse outcomes in the presence of LNM. In several other solid tumours, ENE is recognised as a prognostic factor in the TNM staging system, and we recommend to also include ENE as a prognostic factor in the nodal stage for CRC.^1^ With regard to TD, their association with poor survival has been widely established, and in this meta‐analysis we have shown that LNM + TD+ cases have the worst outcome when compared to LNM+, and even show a trend towards worse outcomes when compared to LNM + ENE+ cases.^2^, ^3^, ^21^ Therefore, we recommend to provide TD with a more prominent position in the nodal stage in the TNM classification and regard them at least as equal, if not more important, than LNM.

When considering how to give TD a more prominent role in nodal staging, it is crucial to address the ‘counting principle’. The current meta‐analysis focuses upon a present versus not present comparison, as we are limited by the available literature data. However, the addition of the number of TD added to the number of LNM (i.e. the counting principle) show improved prognostic accuracy.^27^, ^28^, ^29^ This was confirmed in *post‐hoc* analyses of clinical trials.^30^, ^31^ Therefore, to improve prognostic accuracy TD should be included with LNM count.

In conclusion, this study has found a consistent trend towards worse survival for LNM + TD+ cases compared to LNM + ENE+ cases for DFS, DSS and OS, which was no longer statistically significant in the multivariable meta‐analysis for DFS. Furthermore, both LNM + ENE+ and LNM + TD+ cases have worse survival compared with LNM alone. This provides a more comprehensive understanding of how to view the different types of tumour nodules in the context of locoregional spread in CRC. To improve the accuracy of the staging of CRC patients, we therefore recommend to provide a more important role for both ENE and TD in the nodal stage in the TNM classification, with the most prominent role for TD.

## Conflicts of interest

The authors declare no conflicts of interest.

## Supporting information


**Table S1.** Literature search criteria.
**Table S2.** Quality of the reporting of the included studies.
**Table S3.** Mixed effect estimates from the network meta‐analyses for the different multivariable survival outcomes. Significant hazard ratios are coloured blue and not significant hazard ratios are coloured orange.
**Figure S1.** Forest plots showing direct and indirect evidence for network analyses.
**Figure S2.** Funnel plots for network meta‐analyses.
**Figure S3.** Network comparisons for the network meta‐analysis.
**Figure S4.** Bar graph showing the *P*‐scores for the different groups and for all the different outcome measures. The *P*‐score ranks all compared groups where a higher *P*‐score means a stronger association with worse survival.
**Figure S5.** Direct comparisons hazard ratios.

## Data Availability

Data are available from the corresponding author upon reasonable request.

## References

[1] International Union Against Cancer . Brierley JD , Gospodarowicz MK , Wittekind CH eds. TNM classification of malignant tumours. 8th ed. Hoboken, NJ: Wiley‐Blackwell, 2017.

[2] Lord AC , D'Souza N , Pucher PH *et al*. Significance of extranodal tumour deposits in colorectal cancer: a systematic review and meta‐analysis. Eur. J. Cancer 2017; 82; 92–102. 28651160 10.1016/j.ejca.2017.05.027

[3] Nagtegaal ID , Knijn N , Hugen N *et al*. Tumor deposits in colorectal cancer: improving the value of modern staging‐a systematic review and meta‐analysis. J. Clin. Oncol. 2017; 35; 1119–1127. 28029327 10.1200/JCO.2016.68.9091

[4] Veronese N , Nottegar A , Pea A *et al*. Prognostic impact and implications of extracapsular lymph node involvement in colorectal cancer: a systematic review with meta‐analysis. Ann. Oncol. 2016; 27; 42–48. 26483050 10.1093/annonc/mdv494

[5] Brouwer NPM , Nagtegaal ID . Tumor deposits improve staging in colon cancer: what are the next steps? Ann. Oncol. 2021; 32; 1209–1211. 34416364 10.1016/j.annonc.2021.08.1751

[6] Gabriel W , Dukes C , Bussey H . Lymphatic spread in cancer of the rectum. J. Br. Surg. 1935; 23; 395–413.

[7] International Union Against Cancer . Sobin LH , Gospodarowicz MK , Wittekind CH eds. TNM classification of malignant tumours. 7th ed. Hoboken, NJ: Wiley‐Blackwell, 2010.

[8] Wind J , Lagarde SM , Ten Kate FJ , Ubbink DT , Bemelman WA , van Lanschot JJ . A systematic review on the significance of extracapsular lymph node involvement in gastrointestinal malignancies. Eur. J. Surg. Oncol. 2007; 33; 401–408. 17175130 10.1016/j.ejso.2006.11.001

[9] Chen H , Tang Z , Liu F . Tumor deposit versus extra nodal extension: a differential evaluation of prognostic relevance. Eur. J. Cancer 2018; 105; 127–128. 30409507 10.1016/j.ejca.2018.07.316

[10] Rouse B , Chaimani A , Li T . Network meta‐analysis: an introduction for clinicians. Intern. Emerg. Med. 2017; 12; 103–111. 27913917 10.1007/s11739-016-1583-7 PMC5247317

[11] Hutton B , Salanti G , Caldwell DM *et al*. The PRISMA extension statement for reporting of systematic reviews incorporating network meta‐analyses of health care interventions: checklist and explanations. Ann. Intern. Med. 2015; 162; 777–784. 26030634 10.7326/M14-2385

[12] Brouwer NPM , Lord AC , Terlizzo M *et al*. Interobserver variation in the classification of tumor deposits in rectal cancer‐is the use of histopathological characteristics the way to go? Virchows Arch. 2021; 479; 1111–1118. 34480612 10.1007/s00428-021-03197-0 PMC8724135

[13] Royston P , Parmar MK . Flexible parametric proportional‐hazards and proportional‐odds models for censored survival data, with application to prognostic modelling and estimation of treatment effects. Stat. Med. 2002; 21; 2175–2197. 12210632 10.1002/sim.1203

[14] Knijn N , Nagtegaal ID . Guidelines for reporting histopathology studies. J. Clin. Pathol. 2015; 68; 173–174. 25605774 10.1136/jclinpath-2014-202647corr1

[15] Knijn N , Simmer F , Nagtegaal ID . Recommendations for reporting histopathology studies: a proposal. Virchows Arch. 2015; 466; 611–615. 25846513 10.1007/s00428-015-1762-3 PMC4460276

[16] Higgins JPT , Thomas J , Chandler J *et al*. Cochrane handbook for systematic reviews of interventions. Version 6.4 (updated August 2023) ed. Hoboken, NJ: Cochrane, 2023.

[17] van Valkenhoef G , Dias S , Ades AE , Welton NJ . Automated generation of node‐splitting models for assessment of inconsistency in network meta‐analysis. Res. Synth. Methods 2016; 7; 80–93. 26461181 10.1002/jrsm.1167 PMC5057346

[18] Balduzzi S , Rücker G , Nikolakopoulou A *et al*. netmeta: an R package for network meta‐analysis using frequentist methods. J. Stat. Softw. 2023; 106; 1–40.37138589

[19] Balduzzi S , Rücker G , Schwarzer G . How to perform a meta‐analysis with R: a practical tutorial. Evid. Based Ment. Health 2019; 22; 153–160. 31563865 10.1136/ebmental-2019-300117 PMC10231495

[20] R Development Core Team . R: a language and environment for statistical computing. Vienna, Austria: R Foundation for Statistical Computing, 2023.

[21] Moon JY , Lee MR , Ha GW . Prognostic value of tumor deposits for long‐term oncologic outcomes in patients with stage III colorectal cancer: a systematic review and meta‐analysis. Int. J. Color. Dis. 2022; 37; 141–151. 10.1007/s00384-021-04036-z 34595585

[22] Yamano T , Semba S , Noda M *et al*. Prognostic significance of classified extramural tumor deposits and extracapsular lymph node invasion in T3‐4 colorectal cancer: a retrospective single‐center study. BMC Cancer 2015; 15; 859. 26545360 10.1186/s12885-015-1885-6 PMC4635537

[23] Cao H , Xu E , Liu H , Wan L , Lai M . Epithelial‐mesenchymal transition in colorectal cancer metastasis: a system review. Pathol. Res. Pract. 2015; 211; 557–569. 26092594 10.1016/j.prp.2015.05.010

[24] Brouwer NP , Webbink L , Haddad TS *et al*. Transcriptomics and proteomics reveal distinct biology for lymph node metastases and tumour deposits in colorectal cancer. J. Pathol. 2023; 261; 401–412. 37792663 10.1002/path.6196

[25] Brouwer NPM , Khan A , Bokhorst JM *et al*. The complexity of shapes; how the circularity of tumor nodules impacts prognosis in colorectal cancer. Mod. Pathol. 2023; 37; 100376. 37926423 10.1016/j.modpat.2023.100376

[26] IntHout J , Ioannidis JP , Borm GF , Goeman JJ . Small studies are more heterogeneous than large ones: a meta‐meta‐analysis. J. Clin. Epidemiol. 2015; 68; 860–869. 25959635 10.1016/j.jclinepi.2015.03.017

[27] Li J , Yang S , Hu J *et al*. Tumor deposits counted as positive lymph nodes in TNM staging for advanced colorectal cancer: a retrospective multicenter study. Oncotarget 2016; 7; 18269–18279. 26934317 10.18632/oncotarget.7756 PMC4951287

[28] Pei JP , Zhang CD , Fu X *et al*. A modified tumor‐node‐metastasis classification for stage III colorectal cancers based on treating tumor deposits as positive lymph nodes. Front. Med. (Lausanne) 2020; 7; 571154. 33178717 10.3389/fmed.2020.571154 PMC7593244

[29] Pei JP , Zhang CD , Liang Y *et al*. A modified pathological N stage including status of tumor deposits in colorectal cancer with nodal metastasis. Front. Oncol. 2020; 10; 548692. 33262940 10.3389/fonc.2020.548692 PMC7686583

[30] Cohen R , Shi Q , Meyers J *et al*. Combining tumor deposits with the number of lymph node metastases to improve the prognostic accuracy in stage III colon cancer: a post hoc analysis of the CALGB/SWOG 80702 phase III study (Alliance). Ann. Oncol. 2021; 32; 1267–1275. 34293461 10.1016/j.annonc.2021.07.009 PMC8719434

[31] Delattre JF , Cohen R , Henriques J *et al*. Prognostic value of tumor deposits for disease‐free survival in patients with stage III colon cancer: a post hoc analysis of the IDEA France phase III trial (PRODIGE‐GERCOR). J. Clin. Oncol. 2020; 38; 1702–1710. 32167864 10.1200/JCO.19.01960

[32] Al Sahaf O , Myers E , Jawad M , Browne TJ , Winter DC , Redmond HP . The prognostic significance of extramural deposits and extracapsular lymph node invasion in colon cancer. Dis. Colon Rectum 2011; 54; 982–988. 21730787 10.1097/DCR.0b013e31821c4944

[33] Ambe PC , Gödde D , Störkel S , Zirngibl H , Bönicke L . Extra nodular metastasis is a poor prognostic factor for overall survival in node‐positive patients with colorectal cancer. Int. J. Color. Dis. 2018; 33; 403–409. 10.1007/s00384-018-2991-0 29520454

[34] Brabender J , Bollschweiler E , Hölscher AH *et al*. The prognostic impact of extracapsular lymph node involvement in rectal cancer patients: implications for staging and adjuvant treatment strategies. Oncol. Lett. 2012; 3; 825–830. 22741001 10.3892/ol.2012.569 PMC3362417

[35] Kim H , Rehman A , Chung Y *et al*. Clinicopathologic significance of Extranodal tumor extension in colorectal adenocarcinoma with regional lymph node metastasis. Gastroenterol. Res. Pract. 2016; 2016; 5620765. 27195006 10.1155/2016/5620765 PMC4853953

[36] Kim CW , Kim J , Park Y *et al*. Prognostic implications of extranodal extension in relation to colorectal cancer location. Cancer Res. Treat. 2019; 51; 1135–1143. 30514068 10.4143/crt.2018.392 PMC6639205

[37] Komori K , Kanemitsu Y , Kimura K *et al*. Detailed stratification of TNM stage III rectal cancer based on the presence/absence of extracapsular invasion of the metastatic lymph nodes. Dis. Colon Rectum 2013; 56; 726–732. 23652746 10.1097/DCR.0b013e318286c518

[38] Li T , Yang Y , Wu W *et al*. Prognostic implications of ENE and LODDS in relation to lymph node‐positive colorectal cancer location. Transl. Oncol. 2021; 14; 101190. 34403906 10.1016/j.tranon.2021.101190 PMC8367836

[39] Puppa G , Maisonneuve P , Sonzogni A *et al*. Pathological assessment of pericolonic tumor deposits in advanced colonic carcinoma: relevance to prognosis and tumor staging. Mod. Pathol. 2007; 20; 843–855. 17491597 10.1038/modpathol.3800791

[40] Ueno H , Mochizuki H , Tamakuma S . Prognostic significance of extranodal microscopic foci discontinuous with primary lesion in rectal cancer. Dis. Colon Rectum 1998; 41; 55–61. 9510311 10.1007/BF02236896

[41] Wind J , ten Kate FJ , Kiewiet JJ *et al*. The prognostic significance of extracapsular lymph node involvement in node positive patients with colonic cancer. Eur. J. Surg. Oncol. 2008; 34; 390–396. 17614246 10.1016/j.ejso.2007.05.011

[42] Belt EJ , van Stijn MF , Bril H , de Lange‐de Klerk ES , Meijer GA , Meijer S , et al. Lymph node negative colorectal cancers with isolated tumor deposits should be classified and treated as stage III. Ann. Surg. Oncol. 2010;17:3203–3211. 20625841 10.1245/s10434-010-1152-7 PMC2995864

[43] Goldstein NS , Turner JR . Pericolonic tumor deposits in patients with T3N+MO colon adenocarcinomas: markers of reduced disease free survival and intra‐abdominal metastases and their implications for TNM classification. Cancer 2000; 88; 2228–2238. 10820343

[44] Landau MA , Zhu B , Akwuole FN , Pai RK . Histopathological predictors of recurrence in stage III colon cancer: reappraisal of tumor deposits and tumor budding using AJCC8 criteria. Int. J. Surg. Pathol. 2019; 27; 147–158. 29992847 10.1177/1066896918787275

[45] Nagayoshi K , Ueki T , Nishioka Y *et al*. Tumor deposit is a poor prognostic indicator for patients who have stage II and III colorectal cancer with fewer than 4 lymph node metastases but not for those with 4 or more. Dis. Colon Rectum 2014; 57; 467–474. 24608303 10.1097/DCR.0000000000000059

[46] Qiu HB , Chen G , Keshari RP *et al*. The extramural metastasis might be categorized in lymph node staging for colorectal cancer. BMC Cancer 2011; 11; 414. 21943144 10.1186/1471-2407-11-414 PMC3190391

[47] Shimada Y , Takii Y . Clinical impact of mesorectal extranodal cancer tissue in rectal cancer: detailed pathological assessment using whole‐mount sections. Dis. Colon Rectum 2010; 53; 771–778. 20389211 10.1007/DCR.0b013e3181cf7fd8

[48] Tateishi S , Arima S , Futami K *et al*. A clinicopathological investigation of “tumor nodules” in colorectal cancer. Surg. Today 2005; 35; 377–384. 15864419 10.1007/s00595-004-2950-y

[49] Ueno H , Hashiguchi Y , Shimazaki H *et al*. Peritumoral deposits as an adverse prognostic indicator of colorectal cancer. Am. J. Surg. 2014; 207; 70–77. 24112678 10.1016/j.amjsurg.2013.04.009

[50] von Winterfeld M , Hoffmeister M , Ingold‐Heppner B *et al*. Frequency of therapy‐relevant staging shifts in colorectal cancer through the introduction of pN1c in the 7th TNM edition. Eur. J. Cancer 2014; 50; 2958–2965. 25281526 10.1016/j.ejca.2014.09.002

[51] Yabata E , Udagawa M , Okamoto H . Effect of tumor deposits on overall survival in colorectal cancer patients with regional lymph node metastases. J. Rural Med. 2014; 9; 20–26. 25648159 10.2185/jrm.2880 PMC4310051

