# Supplementary material for: Tumour deposits are associated with worse survival than extranodal extension; a network meta‐analysis on tumour nodules in colorectal cancer
Source: Histopathology. 2024 Aug 28;86(4):485–96. doi: 10.1111/his.15301 (PMC11791726; doi:10.1111/his.15301)
Supplement: Supplementary file 1 — Table S1. Literature search criteria. Table S2. Quality of the reporting of the included studies. Table S3. Mixed effect estimates from the network meta‐analyses for the different multivariable survival outcomes. Significant hazard ratios are coloured blue and not significant hazard ratios are coloured orange. Figure S1. Forest plots showing direct and indirect evidence for network analyses. Figure S2. Funnel plots for network meta‐analyses. Figure S3. Network comparisons for the network meta‐analysis. Figure S4. Bar graph showing the P‐scores for the different groups and for all the different outcome measures. The P‐score ranks all compared groups where a higher P‐score means a stronger association with worse survival. Figure S5. Direct comparisons hazard ratios. [file HIS-86-485-s001.docx]

**Table S1**

Literature search criteria

A. Search strategy on PubMed

**("Colorectal Neoplasms"[Mesh] OR Colorectal Neoplas*[tiab] OR Colorectal Tumor*[tiab] OR Colorectal Cancer*[tiab] OR Colorectal Carcinoma*[tiab] OR Colonic Neoplasm*[tiab] OR Colon Neoplasm*[tiab] OR Cancer of Colon[tiab] OR Colon Cancer*[tiab] OR Cancer of the Colon[tiab] OR Colonic Cancer*[tiab] OR Colon Adenocarcinoma*[tiab] OR Colonic Neoplasm*[tiab] OR Colon Neoplasm*[tiab] OR Cancer of Colon[tiab] OR Colon Cancer*[tiab] OR Cancer of the Colon[tiab] OR Colonic Cancer*[tiab] OR Colon Adenocarcinoma*[tiab] OR Sigmoid Neoplasm*[tiab] OR Sigmoid Colon Neoplasm*[tiab] OR Sigmoid Cancer[tiab] OR Sigmoidal Cancer[tiab] OR Sigmoid Colon Cancer[tiab] OR Cancer of Sigmoid[tiab] OR Cancer of the Sigmoid[tiab] OR Colorectal Tumour*[tiab] OR Rectal Neoplasm*[tiab] OR Rectum Neoplasm*[tiab] OR Rectal Tumor*[tiab] OR Rectal Tumour*[tiab] OR Cancer of Rectum[tiab] OR Rectum Cancer*[tiab] OR Rectal Cancer*[tiab] OR Cancer of the Rectum[tiab]) AND ("Extranodal Extension"[Mesh] OR Extracapsular[tiab] OR Extra-capsular[tiab] OR Tumor Deposit*[tiab] OR Tumour Deposit*[tiab] OR Extranodal[tiab] OR Extra-nodal[tiab] OR neoplastic foci[tiab] OR microfoci*[tiab] OR Non-nodal[tiab] OR Nonnodal[tiab] OR tumor Aggregate*[tiab] OR Tumour Aggregate*[tiab]) AND ("Prognosis"[Mesh:NoExp] OR Prognos*[tiab] OR "Proportional Hazards Models"[Mesh] OR Proportional Hazard Model*[tiab] OR Proportional Hazards Model*[tiab] OR Cox Model*[tiab] OR Hazards Model*[tiab] OR Hazard Model*[tiab] OR Cox Proportional Hazards Model*[tiab] OR Overall Survival[tiab] OR Predict*[tiab] OR "Disease-Free Survival"[Mesh] OR Disease-Free Survival[tiab] OR "Progression-Free Survival"[Mesh] OR Progression Free Survival[tiab] OR Event Free Survival[tiab] OR Recurrence Free Survival[tiab])**

B. Search strategy on Embase

(exp colorectal tumor/ OR Colorectal Neoplas*.ti,ab,kf. OR Colorectal Tumor*.ti,ab,kf. OR Colorectal Cancer*.ti,ab,kf. OR Colorectal Carcinoma*.ti,ab,kf. OR Adenomatous Polyposis Col*.ti,ab,kf. OR "Familial Polyposis Syndrome*".ti,ab,kf. OR "Familial Adenomatous Polypos*".ti,ab,kf. OR "Familial Multiple Polypos*".ti,ab,kf. OR "Familial Polyposis of the Colon".ti,ab,kf. OR Hereditary Polyposis Col*.ti,ab,kf. OR "Familial Multiple Polyposis".ti,ab,kf. OR "Familial Polyposis Syndrome".ti,ab,kf. OR "Myh Associated Polypos*".ti,ab,kf. OR Polyposis Col*.ti,ab,kf. OR "Familial Polyposis Col*".ti,ab,kf. OR "Familial Intestinal Polypos*".ti,ab,kf. OR "Adenomatous Intestinal Polypos*".ti,ab,kf. OR Colonic Neoplasm*.ti,ab,kf. OR Colon Neoplasm*.ti,ab,kf. OR Cancer of Colon.ti,ab,kf. OR Colon Cancer*.ti,ab,kf. OR Cancer of the Colon.ti,ab,kf. OR Colonic Cancer*.ti,ab,kf. OR Colon Adenocarcinoma*.ti,ab,kf. OR Colonic Neoplasm*.ti,ab,kf. OR Colon Neoplasm*.ti,ab,kf. OR Cancer of Colon.ti,ab,kf. OR Colon Cancer*.ti,ab,kf. OR Cancer of the Colon.ti,ab,kf. OR Colonic Cancer*.ti,ab,kf. OR Colon Adenocarcinoma*.ti,ab,kf. OR Sigmoid Neoplasm*.ti,ab,kf. OR Sigmoid Colon Neoplasm*.ti,ab,kf. OR Sigmoid Cancer.ti,ab,kf. OR Sigmoidal Cancer.ti,ab,kf. OR Sigmoid Colon Cancer.ti,ab,kf. OR Cancer of Sigmoid.ti,ab,kf. OR Cancer of the Sigmoid.ti,ab,kf. OR Colorectal Cancer*.ti,ab,kf. OR Colorectal Tumor*.ti,ab,kf. OR Colorectal Tumour*.ti,ab,kf. OR Rectal Neoplasm*.ti,ab,kf. OR Rectum Neoplasm*.ti,ab,kf. OR Rectal Tumor*.ti,ab,kf. OR Rectal Tumour*.ti,ab,kf. OR Cancer of Rectum.ti,ab,kf. OR Rectum Cancer*.ti,ab,kf. OR Rectal Cancer*.ti,ab,kf. OR Cancer of the Rectum.ti,ab,kf. ) AND (extranodal extension/ OR Extracapsular.ti,ab,kf. OR Extra-capsular.ti,ab,kf. OR Tumor Deposit*.ti,ab,kf. OR Tumour Deposit*.ti,ab,kf. OR Extranodal.ti,ab,kf. OR Extra-nodal.ti,ab,kf.) AND (exp prognosis/ OR Prognos*.ti,ab,kf. OR proportional hazards model/ OR Proportional Hazard Model*.ti,ab,kf. OR Proportional Hazards Model*.ti,ab,kf. OR Cox Model*.ti,ab,kf. OR Hazards Model*.ti,ab,kf. OR Hazard Model*.ti,ab,kf. OR Cox Proportional Hazards Model*.ti,ab,kf. OR Overall Survival.ti,ab,kf. OR Predict*.ti,ab,kf. OR exp disease free survival/ OR Disease-Free Survival.ti,ab,kf. OR exp progression free survival/ OR Progression Free Survival.ti,ab,kf. OR Event Free Survival.ti,ab,kf.)

C. Search strategy on Web of science

TS = ("Colorectal Neoplasms"[Mesh] OR Colorectal Neoplas* OR Colorectal Tumor* OR Colorectal Cancer* OR Colorectal Carcinoma* OR Adenomatous Polyposis Col* OR "Familial Polyposis Syndrome*" OR "Familial Adenomatous Polypos*" OR "Familial Multiple Polypos*" OR "Familial Polyposis of the Colon" OR Hereditary Polyposis Col* OR "Familial Multiple Polyposis" OR "Familial Polyposis Syndrome" OR "Myh Associated Polypos*" OR Polyposis Col* OR "Familial Polyposis Col*" OR "Familial Intestinal Polypos*" OR "Adenomatous Intestinal Polypos*" OR Colonic Neoplasm* OR Colon Neoplasm* OR Cancer of Colon OR Colon Cancer* OR Cancer of the Colon OR Colonic Cancer* OR Colon Adenocarcinoma* OR Colonic Neoplasm* OR Colon Neoplasm* OR Cancer of Colon OR Colon Cancer* OR Cancer of the Colon OR Colonic Cancer* OR Colon Adenocarcinoma* OR Sigmoid Neoplasm* OR Sigmoid Colon Neoplasm* OR Sigmoid Cancer OR Sigmoidal Cancer OR Sigmoid Colon Cancer OR Cancer of Sigmoid OR Cancer of the Sigmoid OR Colorectal Cancer* OR Colorectal Tumor* OR Colorectal Tumour* OR Rectal Neoplasm* OR Rectum Neoplasm* OR Rectal Tumor* OR Rectal Tumour* OR Cancer of Rectum OR Rectum Cancer* OR Rectal Cancer* OR Cancer of the Rectum) AND TS = ("Extranodal Extension"[Mesh] OR Extracapsular OR Extra-capsular OR Tumor Deposit* OR Tumour Deposit* OR Extranodal OR Extra-nodal) AND TS = ("Prognosis"[Mesh:NoExp] OR Prognos* OR "Proportional Hazards Models"[Mesh] OR Proportional Hazard Model* OR Proportional Hazards Model* OR Cox Model* OR Hazards Model* OR Hazard Model* OR Cox Proportional Hazards Model* OR Overall Survival OR Predict* OR "Disease-Free Survival"[Mesh] OR Disease-Free Survival OR "Progression-Free Survival"[Mesh] OR Progression Free Survival OR Event Free Survival)

D. Search strategy on Cochrane

(([mh "Colorectal Neoplasms"] OR ("Colorectal" NEXT Neoplas*):ti,ab,kw  OR ("Colorectal" NEXT Tumor*):ti,ab,kw  OR ("Colorectal" NEXT Cancer*):ti,ab,kw  OR ("Colorectal" NEXT Carcinoma*):ti,ab,kw  OR ("Adenomatous Polyposis" NEXT Col*):ti,ab,kw  OR ("Familial Polyposis" NEXT Syndrome*):ti,ab,kw  OR ("Familial Adenomatous" NEXT Polypos*):ti,ab,kw  OR ("Familial Multiple" NEXT Polypos*):ti,ab,kw  OR "Familial Polyposis of the Colon":ti,ab,kw  OR ("Hereditary Polyposis" NEXT Col*):ti,ab,kw  OR "Familial Multiple Polyposis":ti,ab,kw  OR "Familial Polyposis Syndrome":ti,ab,kw  OR ("Myh Associated" NEXT Polypos*):ti,ab,kw  OR ("Polyposis" NEXT Col*):ti,ab,kw  OR ("Familial Polyposis" NEXT Col*):ti,ab,kw  OR ("Familial Intestinal" NEXT Polypos*):ti,ab,kw  OR ("Adenomatous Intestinal" NEXT Polypos*):ti,ab,kw  OR ("Colonic" NEXT Neoplasm*):ti,ab,kw  OR ("Colon" NEXT Neoplasm*):ti,ab,kw  OR "Cancer of Colon":ti,ab,kw  OR ("Colon" NEXT Cancer*):ti,ab,kw  OR "Cancer of the Colon":ti,ab,kw  OR ("Colonic" NEXT Cancer*):ti,ab,kw  OR ("Colon" NEXT Adenocarcinoma*):ti,ab,kw  OR ("Colonic" NEXT Neoplasm*):ti,ab,kw  OR ("Colon" NEXT Neoplasm*):ti,ab,kw  OR "Cancer of Colon":ti,ab,kw  OR ("Colon" NEXT Cancer*):ti,ab,kw  OR "Cancer of the Colon":ti,ab,kw  OR ("Colonic" NEXT Cancer*):ti,ab,kw  OR ("Colon" NEXT Adenocarcinoma*):ti,ab,kw  OR ("Sigmoid" NEXT Neoplasm*):ti,ab,kw  OR ("Sigmoid Colon" NEXT Neoplasm*):ti,ab,kw  OR "Sigmoid Cancer":ti,ab,kw  OR "Sigmoidal Cancer":ti,ab,kw  OR "Sigmoid Colon Cancer":ti,ab,kw  OR "Cancer of Sigmoid":ti,ab,kw  OR "Cancer of the Sigmoid":ti,ab,kw  OR ("Colorectal" NEXT Cancer*):ti,ab,kw  OR ("Colorectal" NEXT Tumor*):ti,ab,kw  OR ("Colorectal" NEXT Tumour*):ti,ab,kw  OR ("Rectal" NEXT Neoplasm*):ti,ab,kw  OR ("Rectum" NEXT Neoplasm*):ti,ab,kw  OR ("Rectal" NEXT Tumor*):ti,ab,kw  OR ("Rectal" NEXT Tumour*):ti,ab,kw  OR "Cancer of Rectum":ti,ab,kw  OR ("Rectum" NEXT Cancer*):ti,ab,kw  OR ("Rectal" NEXT Cancer*):ti,ab,kw  OR "Cancer of the Rectum":ti,ab,kw ) AND ([mh "Extranodal Extension"] OR Extracapsular:ti,ab,kw  OR Extra-capsular:ti,ab,kw  OR ("Tumor" NEXT Deposit*):ti,ab,kw  OR ("Tumour" NEXT Deposit*):ti,ab,kw  OR Extranodal:ti,ab,kw  OR Extra-nodal:ti,ab,kw )) AND ([mh ^Prognosis] OR Prognos*:ti,ab,kw  OR [mh "Proportional Hazards Models"] OR ("Proportional Hazard" NEXT Model*):ti,ab,kw  OR ("Proportional Hazards" NEXT Model*):ti,ab,kw  OR ("Cox" NEXT Model*):ti,ab,kw  OR ("Hazards" NEXT Model*):ti,ab,kw  OR ("Hazard" NEXT Model*):ti,ab,kw  OR ("Cox Proportional Hazards" NEXT Model*):ti,ab,kw  OR "Overall Survival":ti,ab,kw  OR Predict*:ti,ab,kw  OR [mh "Disease-Free Survival"] OR "Disease-Free Survival":ti,ab,kw  OR [mh "Progression-Free Survival"] OR "Progression Free Survival":ti,ab,kw  OR "Event Free Survival":ti,ab,kw )

**Table S2**

Quality of the reporting of the included studies

|  |  | Al Sahaf | Ambe | Belt | Brabender | Goldstein | Kim | Kim | Komori | Landau | Li | Nagayoshi | Puppa | Qiu | Shimada | Tateishi | Ueno | Ueno | Wind | Winterfeld | Yabata |
| --- | --- | --- | --- | --- | --- | --- | --- | --- | --- | --- | --- | --- | --- | --- | --- | --- | --- | --- | --- | --- | --- |
|  |  | 2011 | 2018 | 2010 | 2012 | 2000 | 2016 | 2019 | 2013 | 2019 | 2021 | 2014 | 2007 | 2011 | 2010 | 2001 | 2014 | 1998 | 2008 | 2014 | 2014 |
| No. | Criterion | TD & ENE | ENE | TD | ENE | TD | TD & ENE | ENE | ENE | TD | ENE | TD | TD & ENE | TD | TD | TD | TD | ENE | ENE | TD | TD |
| 1 | States the factor of interest, study objectives and hypothesis | 1 | 1 | 1 | 1 | 1 | 1 | 1 | 1 | 1 | 1 | 1 | 1 | 1 | 1 | 1 | 1 | 1 | 1 | 1 | 1 |
| 2 | Describes patient characteristics, inclusion and exclusion criteria | 1 | 1 | 1 | 1 | 1 | 1 | 1 | 1 | 1 | 1 | 1 | 1 | 1 | 1 | 1 | 0 | 1 | 1 | 1 | 1 |
| 3 | Describes preoperative treatment details (NA in studies about colon carcinomas) | NA | 0 | 1 | 1 | NA | 1 | 1 | 1 | NA | 1 | 1 | 1 | 0 | 1 | 0 | 1 | 0 | NA | 1 | 1 |
| 4 | Describes the number of slides examined for TDs/ENE | 0 | 0 | 1 | 1 | 1 | 1 | 1 | 0 | 1 | 1 | 1 | 1 | 1 | 1 | 1 | 1 | 1 | 1 | 1 | 0 |
| 5 | Specifies criteria for TD/ENE | 1 | 0 | 1 | 1 | 1 | 1 | 1 | 1 | 1 | 1 | 1 | 1 | 1 | 1 | 1 | 0 | 1 | 1 | 1 | 1 |
| 6 | Defines how far TDs should be located from tumor (prevent direct growth) (NA in studies about ENE) | 0 | NA | 0 | NA | 0 | NA | NA | NA | 0 | 0 | 1 | 0 | 0 | 0 | 0 | 1 | 0 | 0 | 0 | 0 |
| 7 | Mentions location (minimally rectum/colon) | 1 | 1 | 1 | 1 | 1 | 1 | 1 | 1 | 1 | 1 | 1 | 1 | 1 | 1 | 1 | 1 | 1 | 1 | 1 | 1 |
| 8 | Describes the number of independent blinded scorers | 1 | 1 | 1 | 0 | 0 | 1 | 1 | 0 | 0 | 0 | 0 | 1 | 1 | 1 | 0 | 1 | 0 | 1 | 1 | 1 |
| 9 | Mentions the hospital where the samples came from | 1 | 1 | 1 | 1 | 1 | 1 | 1 | 1 | 1 | 0 | 1 | 1 | 1 | 1 | 1 | 1 | 1 | 1 | 1 | 1 |
| 10 | Mentions the timeframe of included samples | 0 | 1 | 1 | 1 | 1 | 1 | 1 | 1 | 1 | 1 | 1 | 1 | 1 | 1 | 1 | 1 | 1 | 1 | 1 | 1 |
| 11 | Defines DFS (N/A in studies that did not perform DFS analysis) | 0 | NA | 1 | NA | 1 | 1 | 0 | 0 | 1 | 1 | 0 | 1 | 0 | 0 | NA | 0 | NA | 0 | 0 | 1 |
| 12 | Defines DSS (N/A in studies that did not perform DSS analysis) | 1 | NA | NA | NA | NA | NA | NA | NA | NA | NA | NA | 1 | NA | NA | 0 | 1 | NA | NA | 0 | NA |
| 13 | Defines OS (N/A in studies that did not perform OS analysis) | NA | 0 | NA | 0 | NA | NA | 0 | 0 | NA | 0 | 0 | NA | NA | 0 | NA | NA | 0 | NA | 0 | 1 |
| 14 | Describes end of follow-up period/data (N/A in studies that did not perform outcome analysis) | 1 | 0 | 0 | 0 | 0 | 0 | 0 | 0 | 0 | 1 | 0 | 0 | 1 | 0 | 0 | 0 | 0 | 1 | 1 | 0 |
| 15 | Reports median follow-up time (N/A in studies that did not perform outcome analysis) | 1 | 0 | 1 | 1 | 1 | 0 | 1 | 0 | 1 | 1 | 1 | 0 | 0 | 1 | 0 | 1 | 0 | 1 | 0 | 0 |
| 16 | Describes number of patients included in the analysis and reason for dropout | 1 | 0 | 1 | 1 | 1 | 1 | 1 | 1 | 1 | 1 | 1 | 1 | 1 | 1 | 0 | 1 | 1 | 1 | 1 | 1 |
| 17 | Reports patient characteristics (at least T stage, N stage, M stage) | 0 | 1 | 1 | 1 | 1 | 1 | 1 | 1 | 1 | 1 | 1 | 1 | 1 | 1 | 0 | 1 | 0 | 1 | 1 | 1 |
| 18 | Reports the relation of TDs/ENE to standard prognostic variables | 1 | 0 | 1 | 1 | 0 | 1 | 1 | 1 | 1 | 1 | 1 | 1 | 1 | 1 | 1 | 1 | 0 | 1 | 1 | 1 |
| 19 | >90% of initial cases includes in the UV/MV analysis (NA in studies that did not perform outcome analysis) | 0 | NA | 0 | 1 | 0 | 1 | 0 | 0 | 0 | 1 | 1 | 1 | 0 | 1 | NA | 1 | NA | 1 | 1 | 1 |
| 20 | Reports the estimated effect (HR/RR, CI, P value; freq in table) for TDs/ENE on survival in the UV analysis (NA in studies that did not perform outcome analysis) | 1 | NA | 0 | 0 | 0 | 0 | 1 | 0 | 1 | 0 | 1 | 1 | 1 | 0 | NA | 1 | NA | 0 | 0 | 0 |
| 21 | Reports the estimated effect (HR, CI, P value) for TDs/ENE on survival in MV analysis (NA in studies that did not perform outcome analysis) | 1 | NA | 1 | 1 | 1 | 1 | 1 | 1 | 1 | 1 | 1 | 1 | 1 | 1 | NA | 1 | NA | 1 | 1 | 1 |
| 22 | Reports the estimated effects of all other prognostic factors included in MV analysis (NA in studies who did not perform outcome analysis) | 1 | NA | 0 | 1 | 0 | 1 | 1 | 1 | 1 | 1 | 1 | 1 | 1 | 1 | NA | 1 | NA | 1 | 0 | 1 |
| 23 | Interprets the results in context of the prespecified hypotheses and other relevant studies; include a discussion of limitations of the study | 1 | 1 | 1 | 1 | 1 | 1 | 1 | 1 | 1 | 1 | 1 | 1 | 1 | 1 | 1 | 1 | 1 | 1 | 1 | 1 |
| 24 | Discusses implications for future research and clinical value | 1 | 1 | 1 | 1 | 1 | 1 | 1 | 1 | 1 | 1 | 1 | 1 | 1 | 1 | 1 | 1 | 1 | 1 | 1 | 1 |
| Total score (% of items reported) | | 73% | 53% | 77% | 81% | 67% | 86% | 82% | 64% | 81% | 78% | 83% | 87% | 77% | 78% | 56% | 83% | 56% | 86% | 71% | 78% |

**Figure S1**

Forest plots showing direct and indirect evidence for network analyses

(A) Forest plot network analysis univariable disease-free survival. (B) Forest plots network analysis multivariable disease-free survival. (C) Forest plot network analysis univariable overall survival. (D) Forest plots network analysis multivariable overall survival. (E) Forest plot network analysis univariable disease-specific survival. (F) Forest plots network analysis multivariable disease-specific survival.

**A**

**B**

**C**


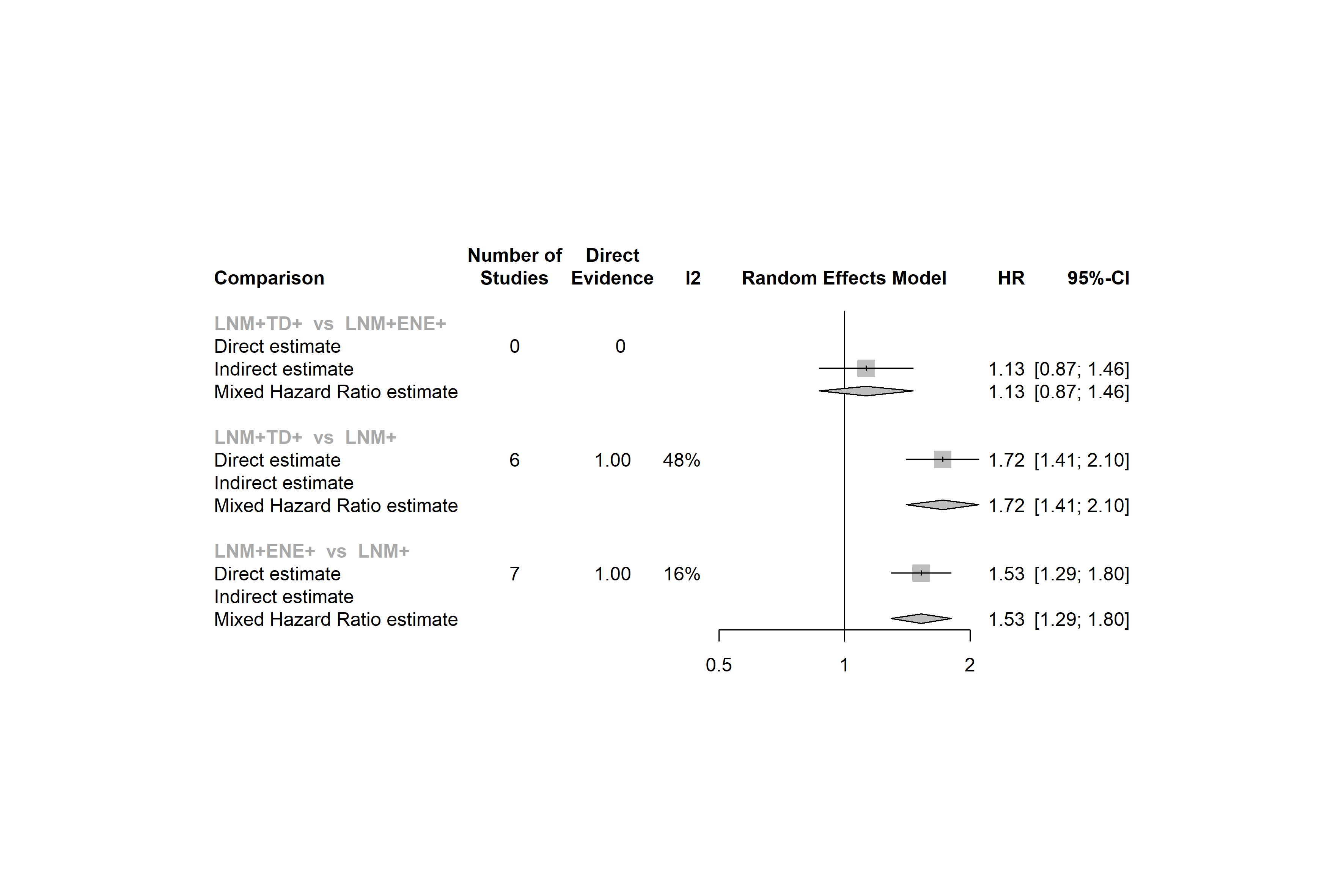


**D**


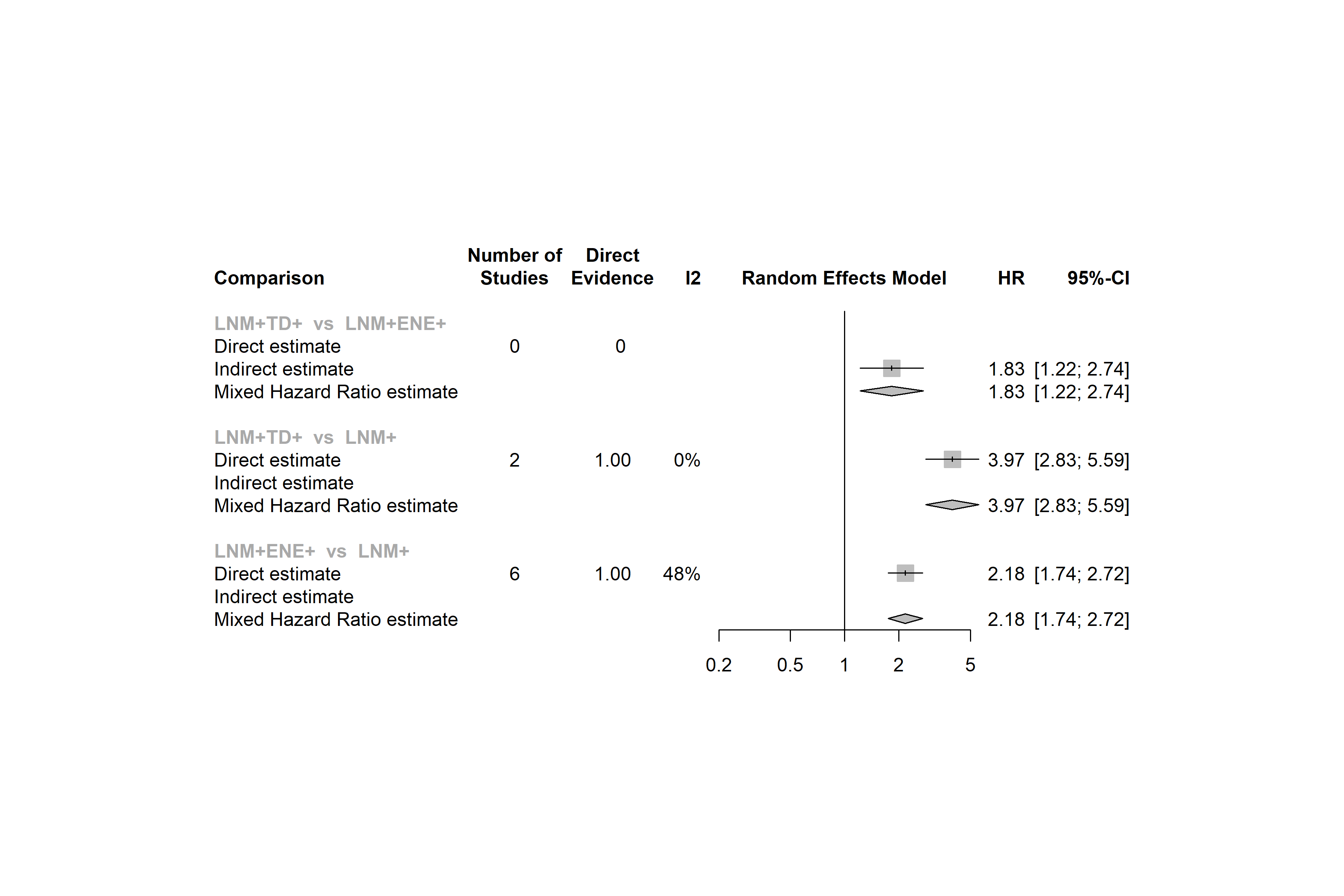

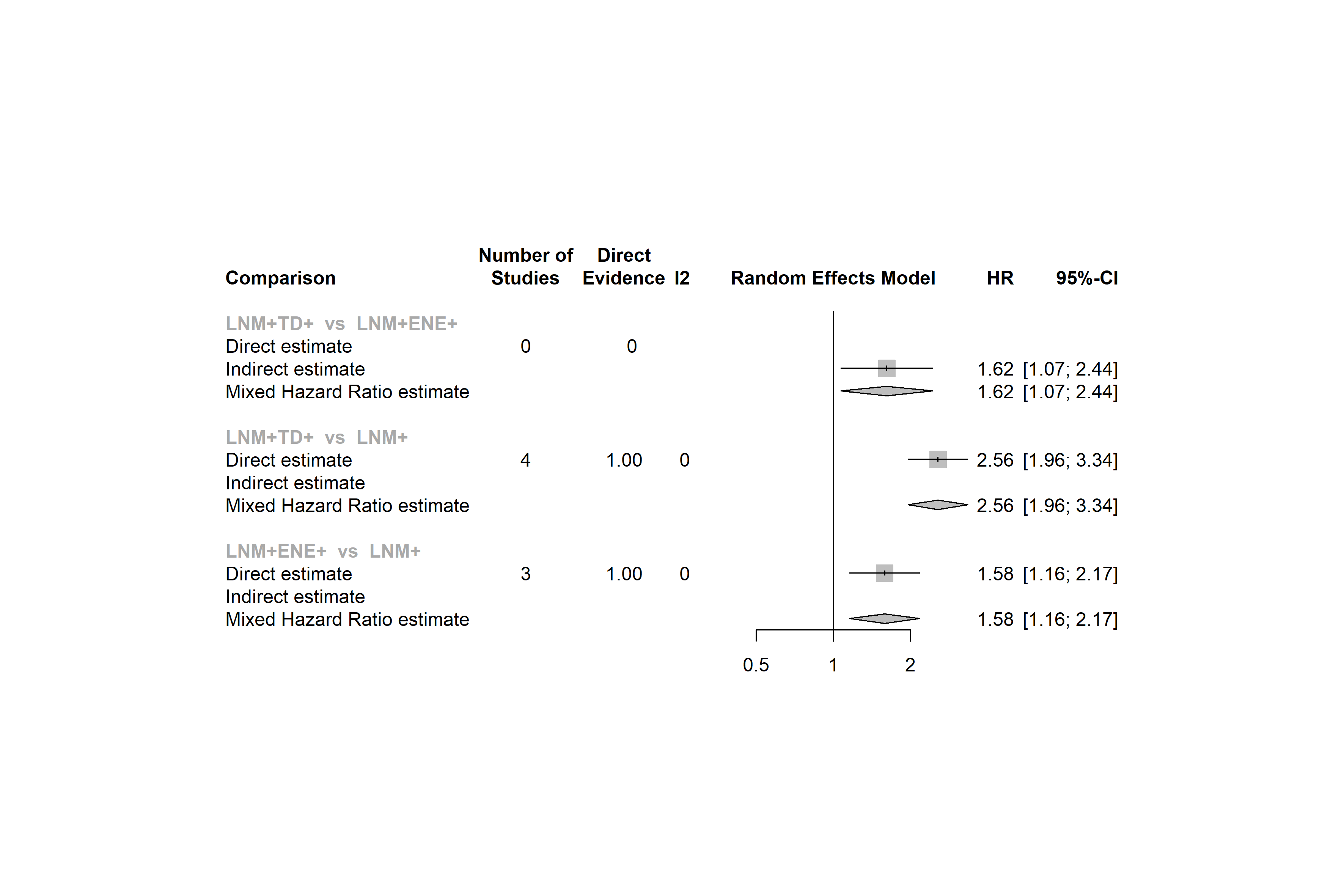

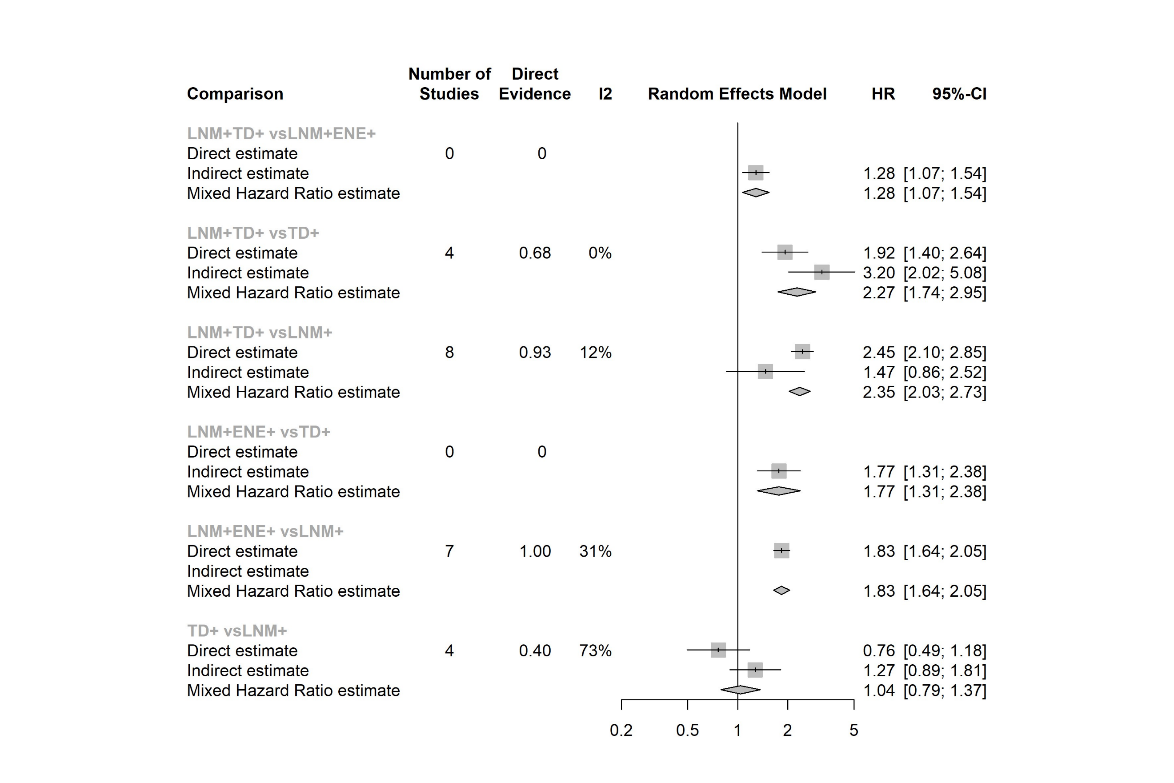


**E**

**F**


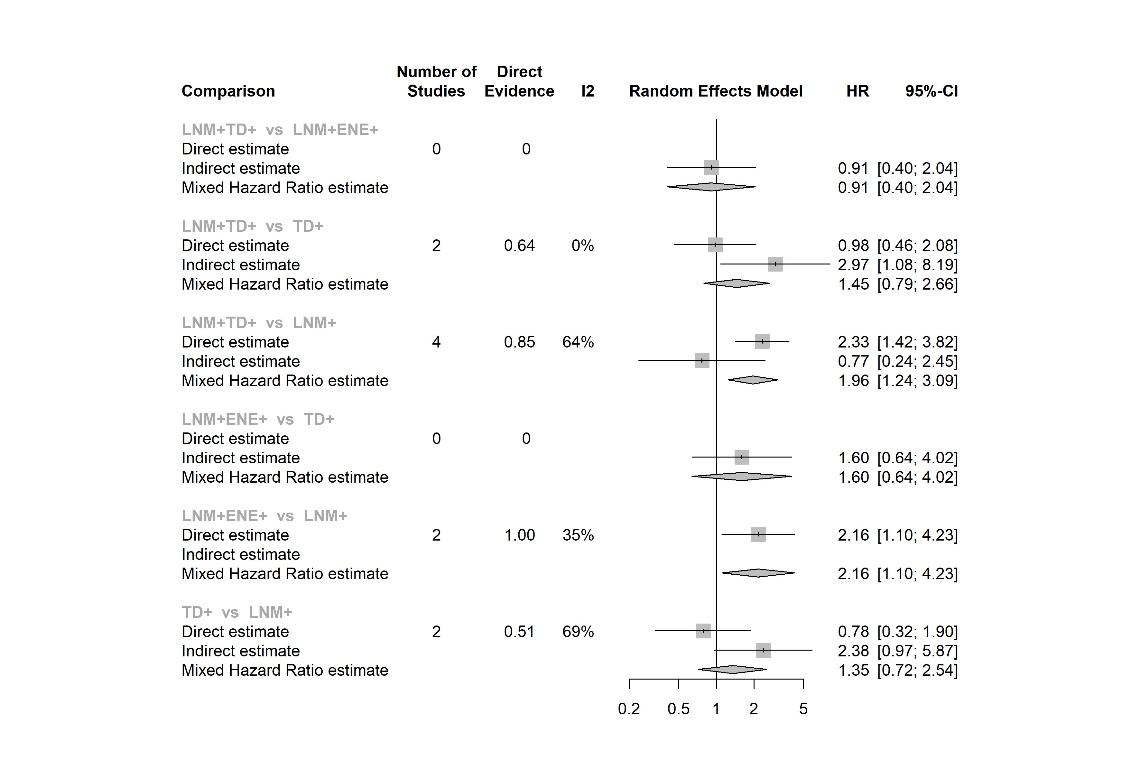

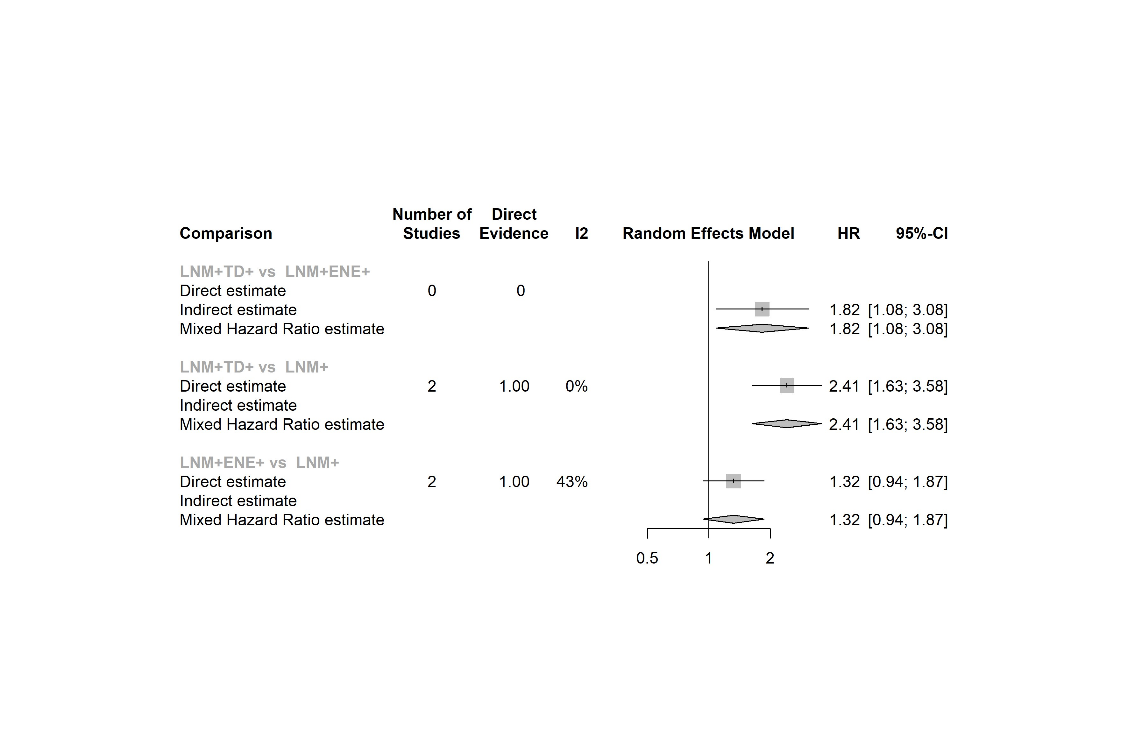


**Figure S2**

Funnel plots for network meta-analyses

(A) Funnel plot network analysis univariable disease-free survival. (B) Funnel plot network analysis multivariable disease-free survival. (C) Funnel plot network analysis univariable overall survival. (D) Funnel plot network analysis multivariable overall survival. (E) Funnel plot network analysis univariable disease-specific survival. (F) Funnel plot network analysis multivariable disease-specific survival.


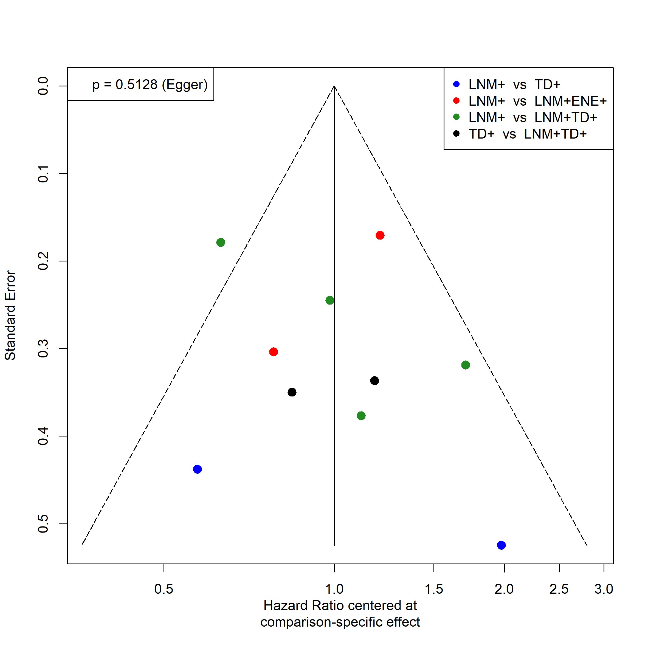


**A**

**B**


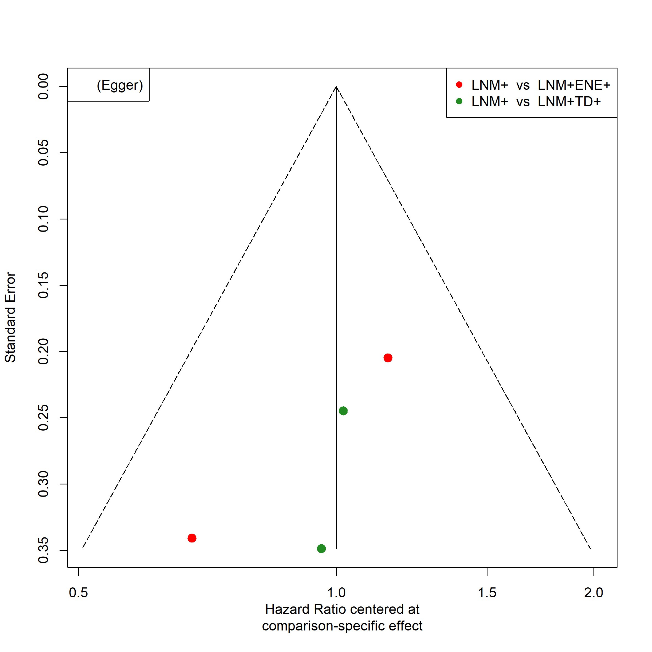


**C**


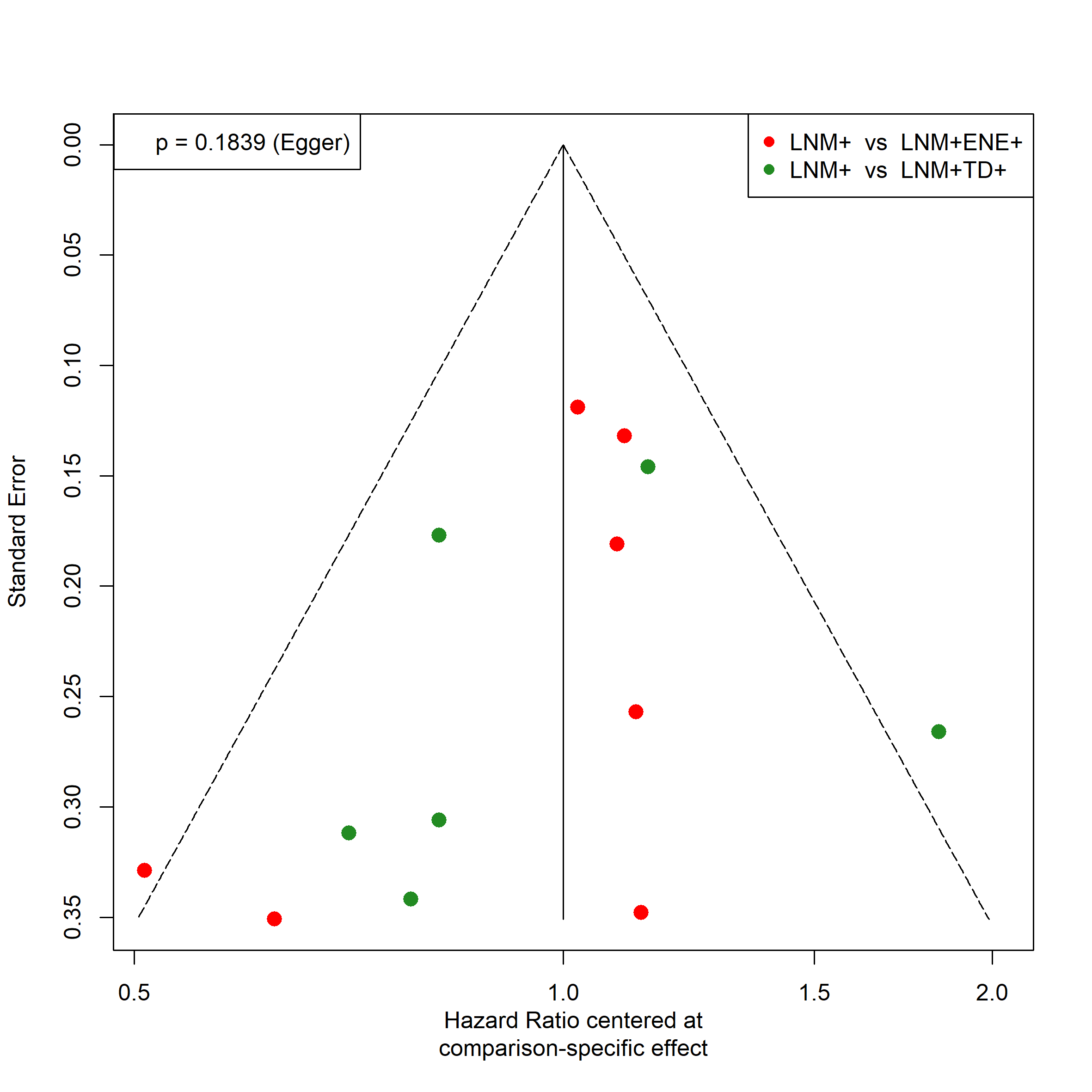


**D**

**F**

**E**


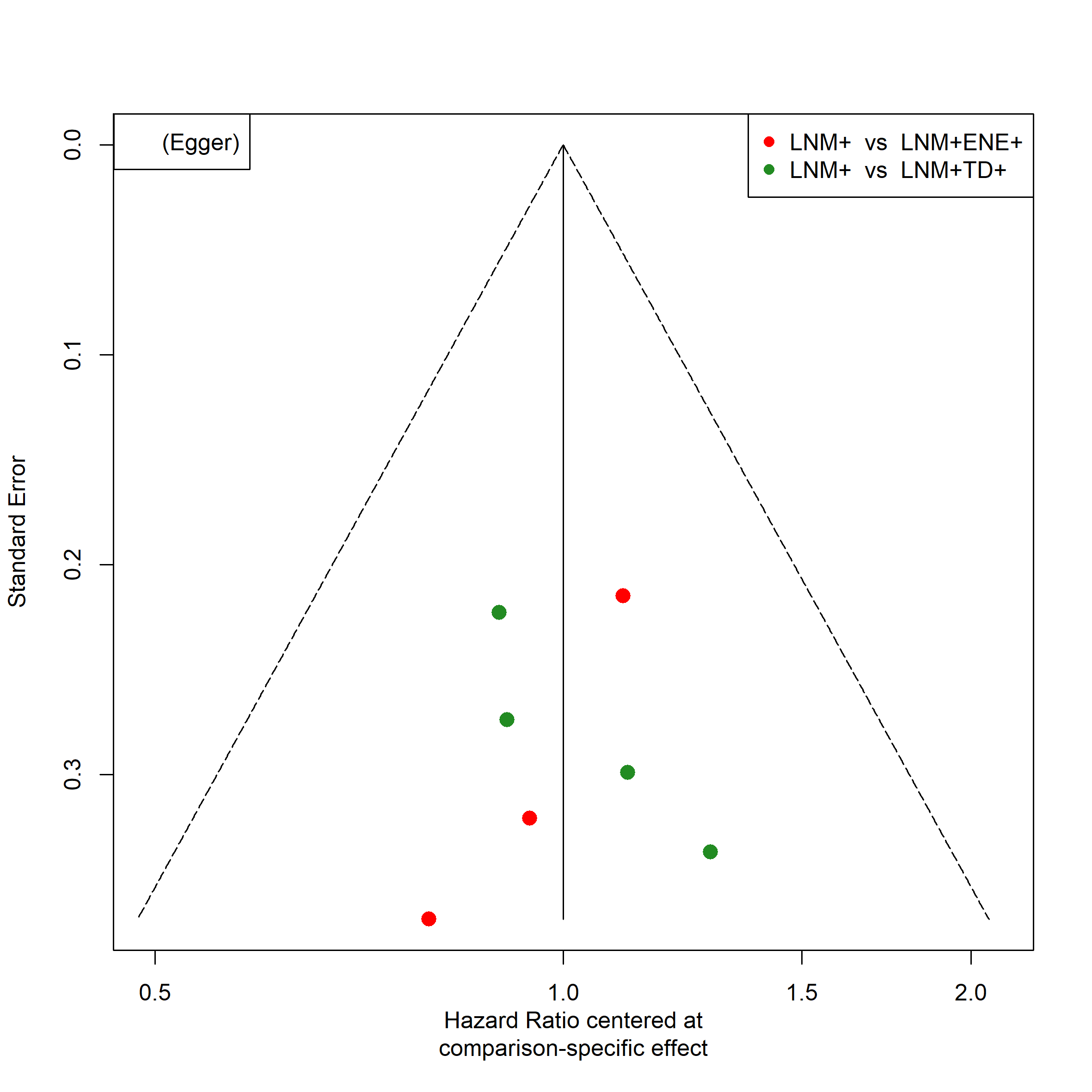

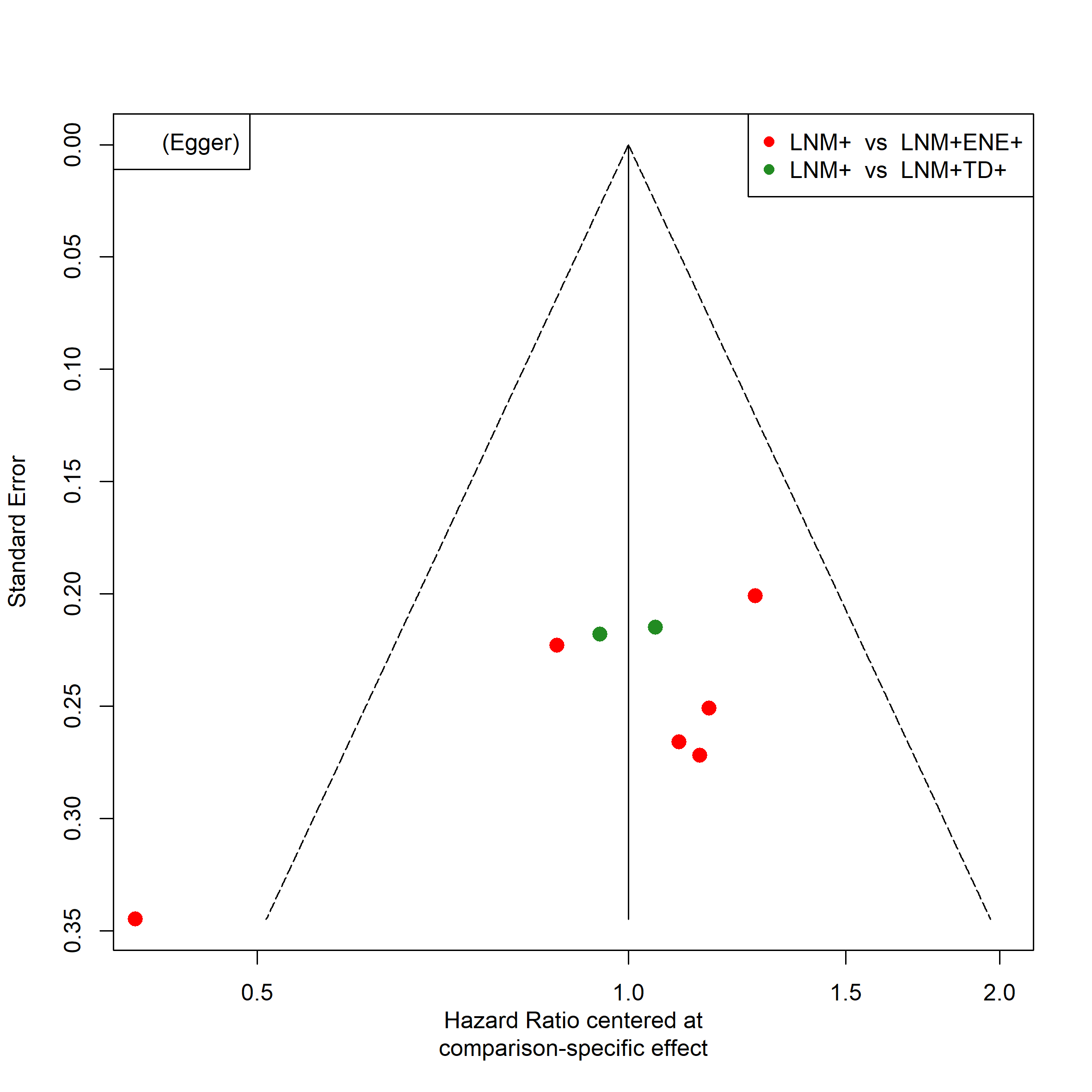

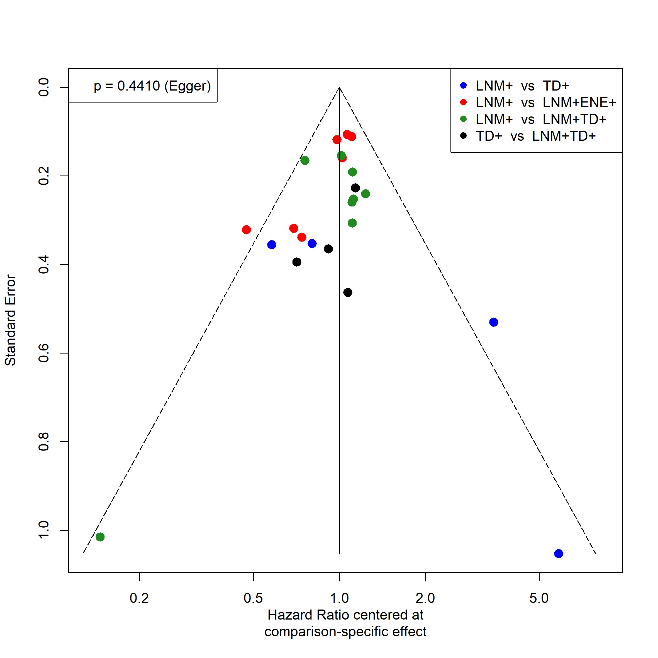


**Figure S3**

Network comparisons for the network meta-analysis.

(A) Network comparisons multivariable disease-free survival. (B) Network comparisons univariable overall survival. (C) Network comparisons multivariable overall survival. (D) Network comparisons univariable disease-specific survival (E) Network comparisons multivariable disease-specific survival.


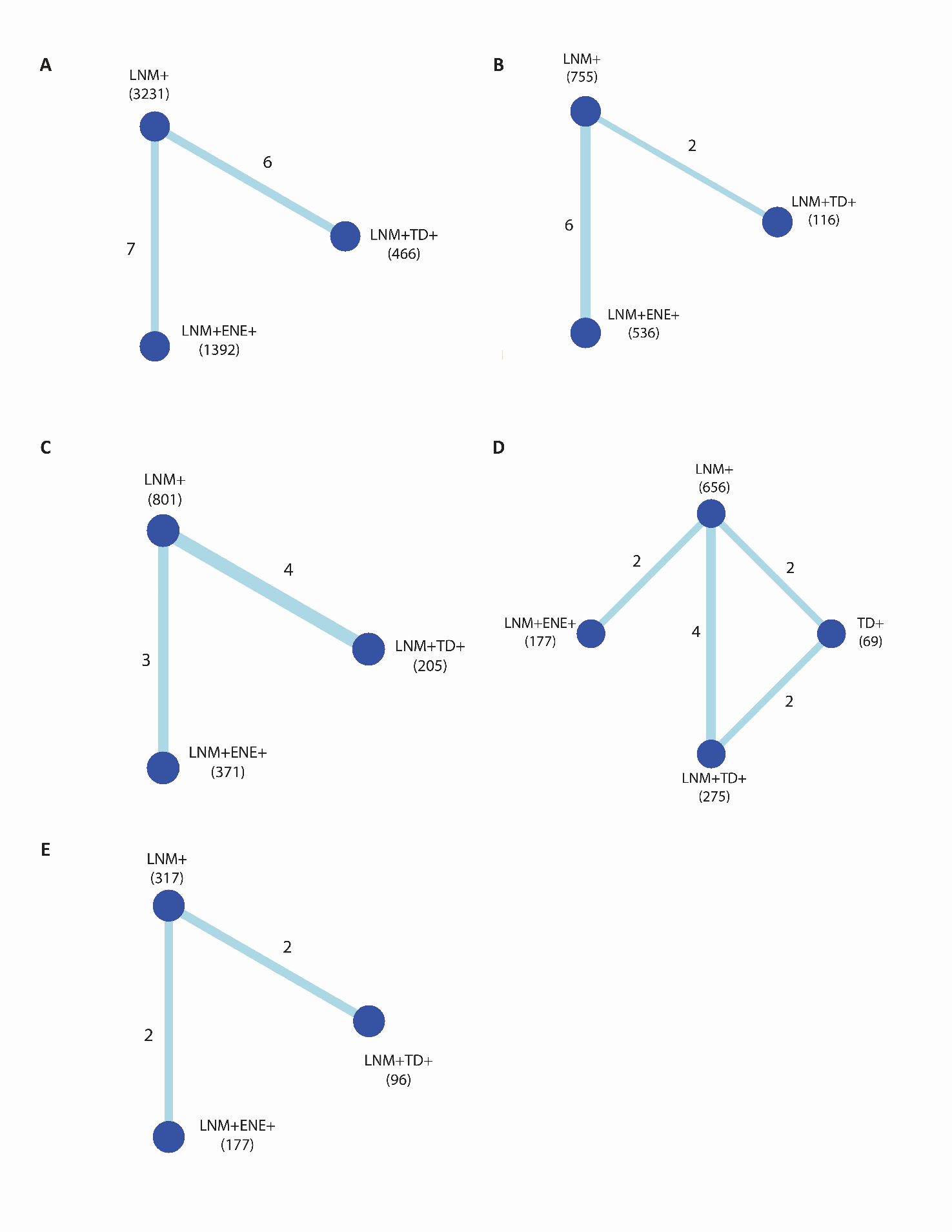


**Figure S4**

Bar graph showing the P-scores for the different groups and for all the different outcome measures. The P-score ranks all compared groups where a higher P-score means a stronger association with worse survival.


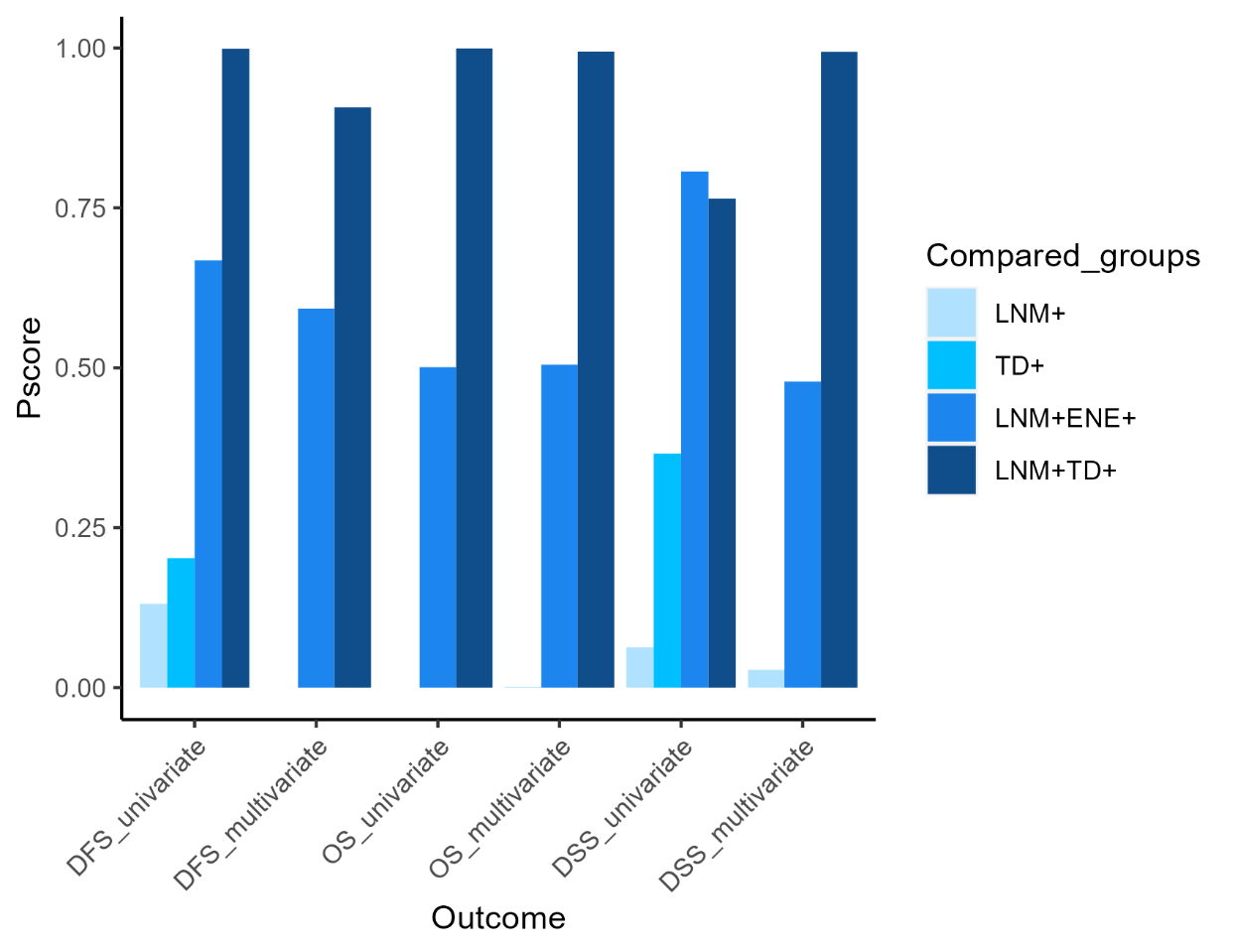


**Figure S5**

Direct comparisons hazard ratios.

(A) Direct comparisons of multivariable disease-free survival. (B) Direct comparisons of univariable disease-specific survival. (C) Direct comparisons of multivariable disease-specific survival. (D) Direct comparisons of univariable overall survival. (E) Direct comparisons of multivariable overall survival.

**A**

*Disease-free survival - LNM+TD+ vs LNM+ (multivariable)*

*Disease-free survival - LNM+ENE+ vs LNM+ (multivariable)*


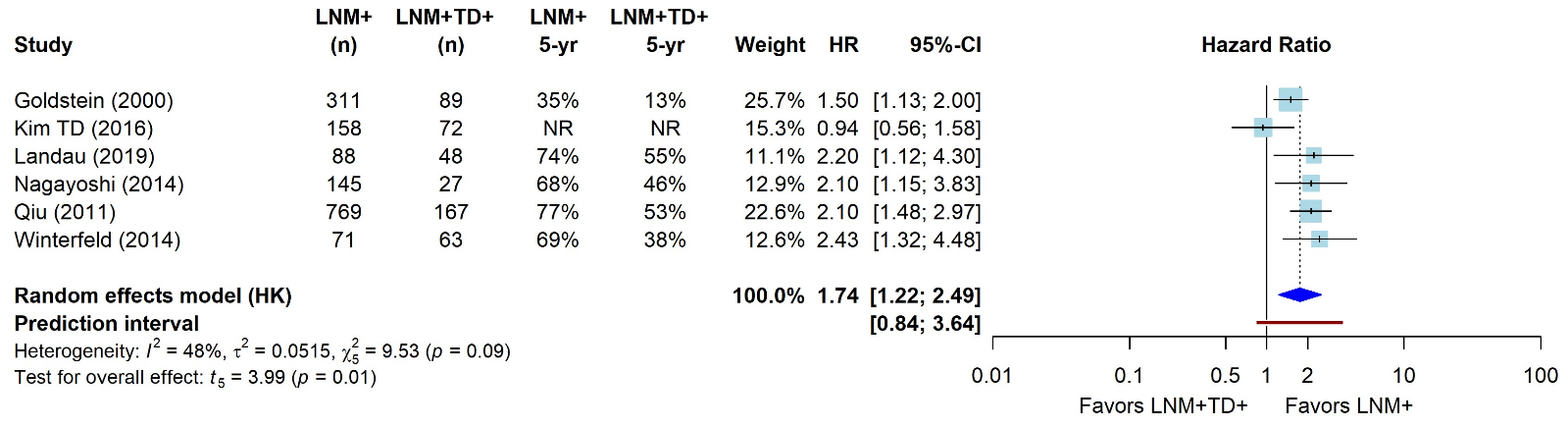

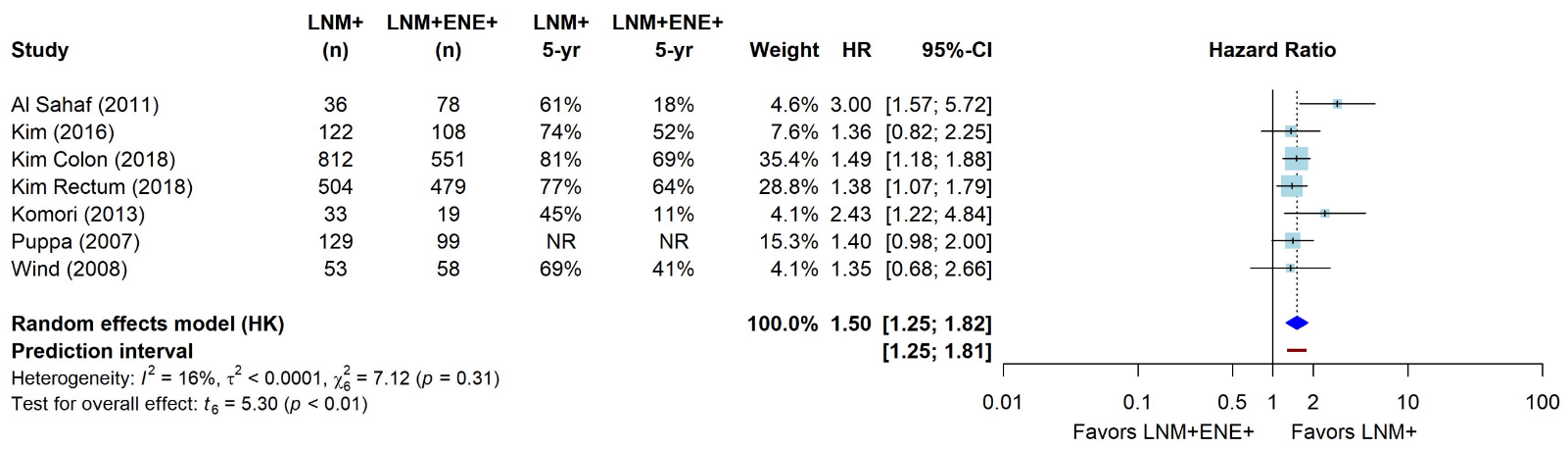


**B**

*Disease-specific survival - LNM+TD+ vs LNM+ (univariable)*

*Disease-specific survival - LNM+ENE+ vs LNM+ (univariable)*

*Disease-specific survival - LNM+ vs TD+ (univariable)*

*Disease-specific survival - LNM+TD+ vs TD+ (univariable)*


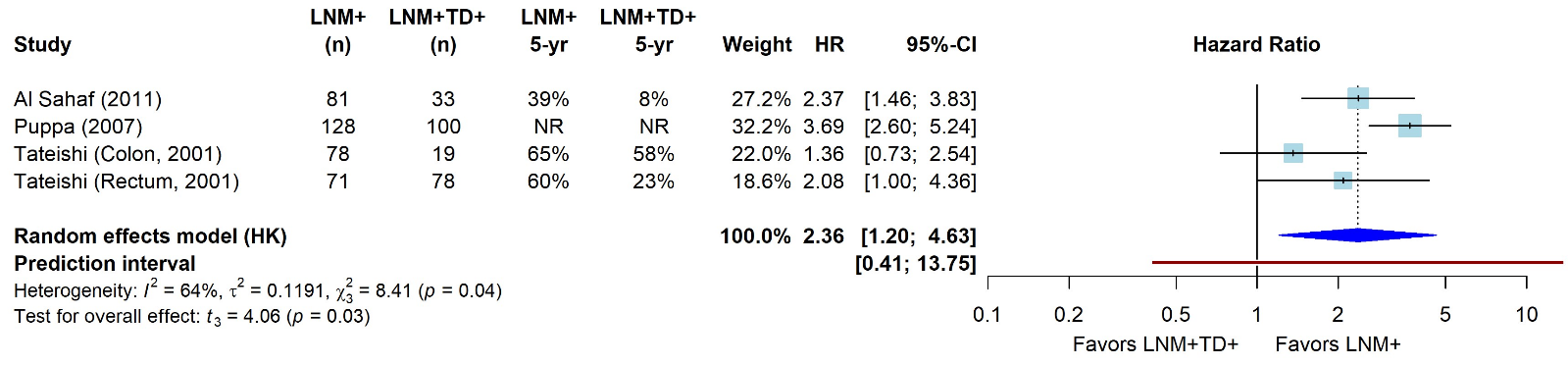

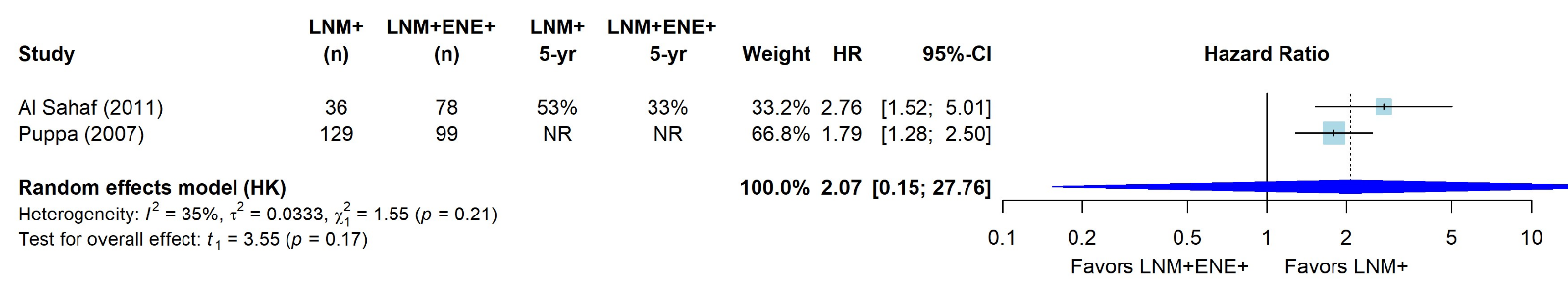

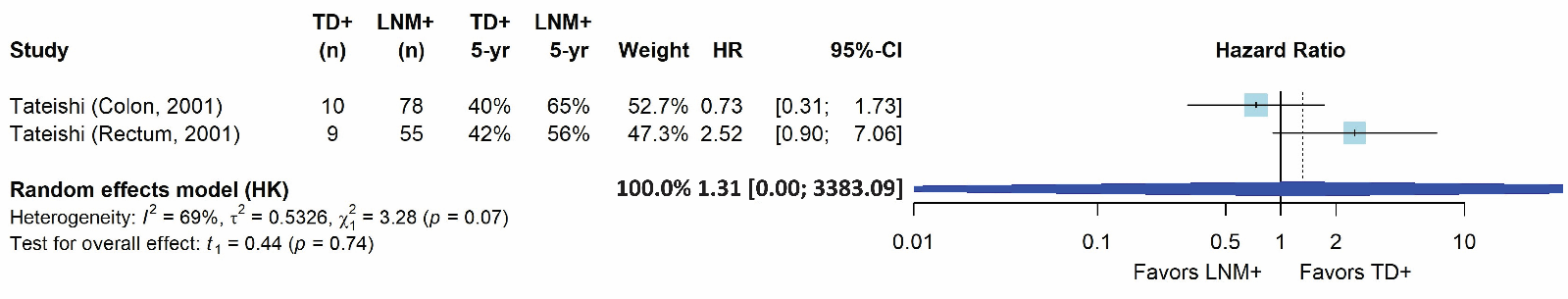

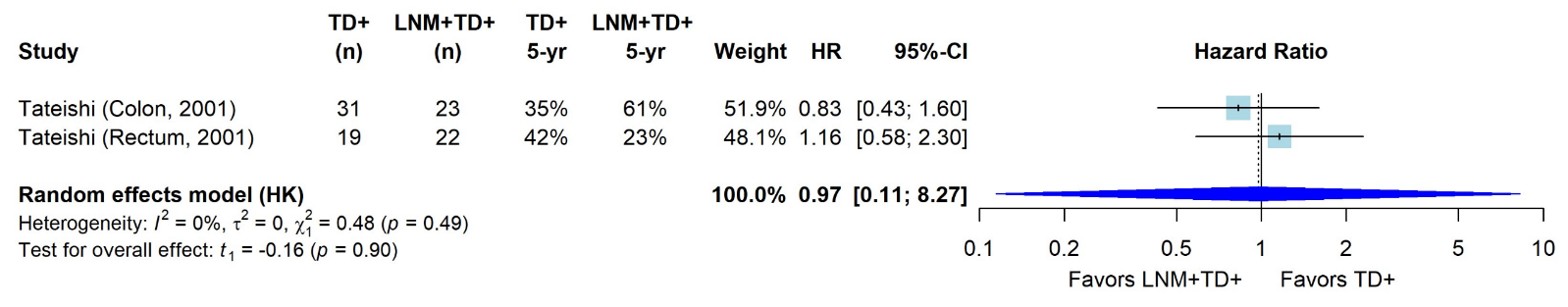


**D**

*Overall survival - LNM+TD+ vs LNM+ (univariable)*

*Overall survival - LNM+ENE+ vs LNM+ (univariable)*


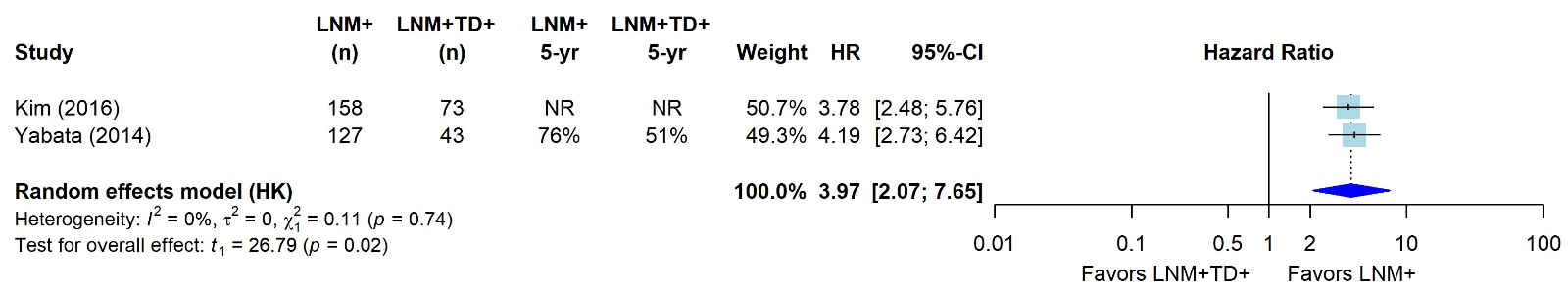

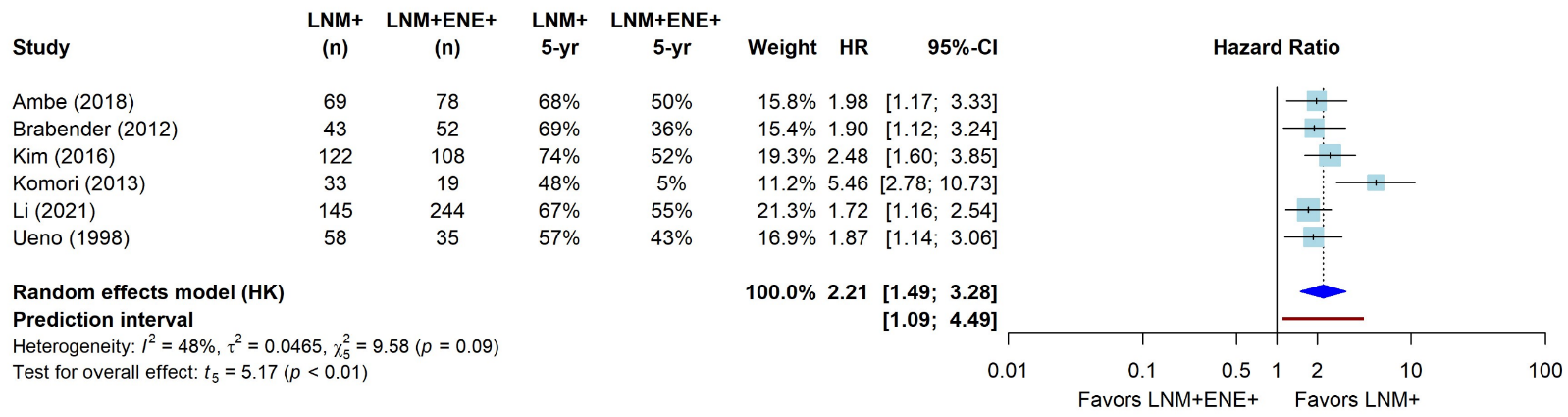


**C**

*Disease-specific survival - LNM+TD+ vs LNM+ (multivariable)*

*Disease-specific survival - LNM+ENE+ vs LNM+ (multivariable)*


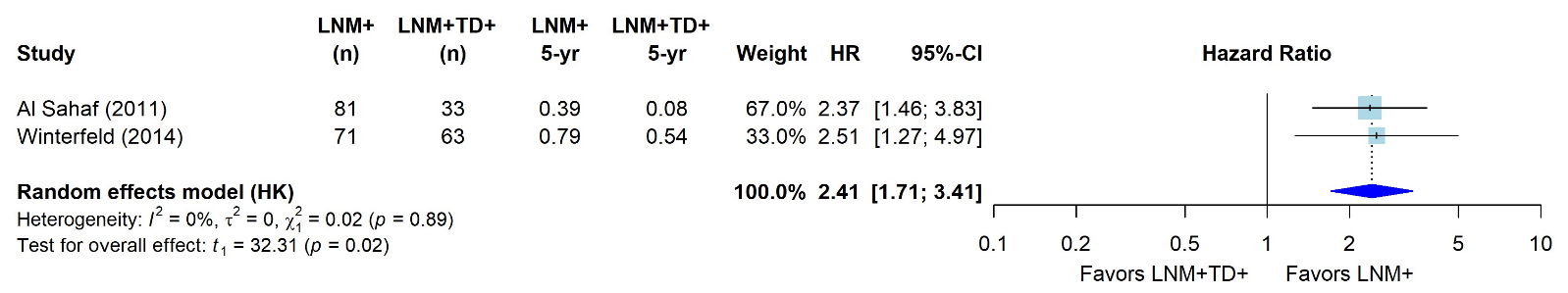

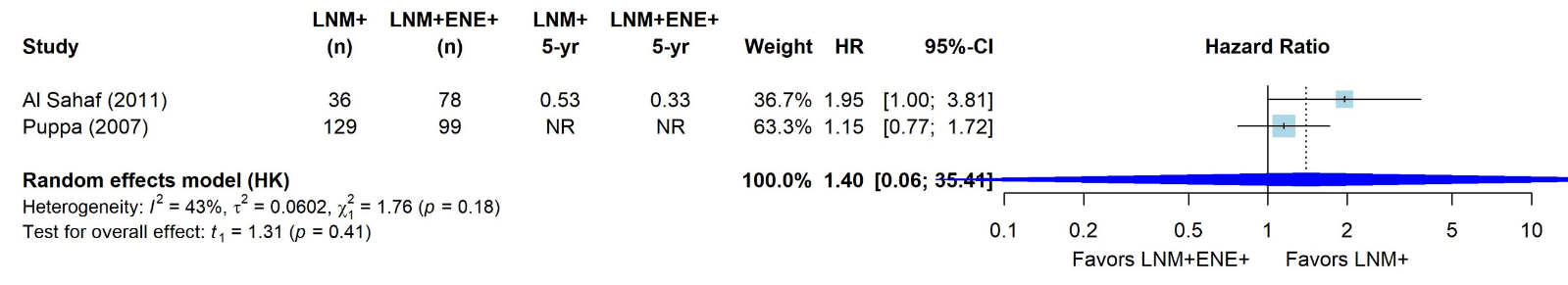


*Overall survival - LNM+TD+ vs LNM+ (multivariable)*

*Overall survival - LNM+ENE+ vs LNM+ (multivariable)*

**E**


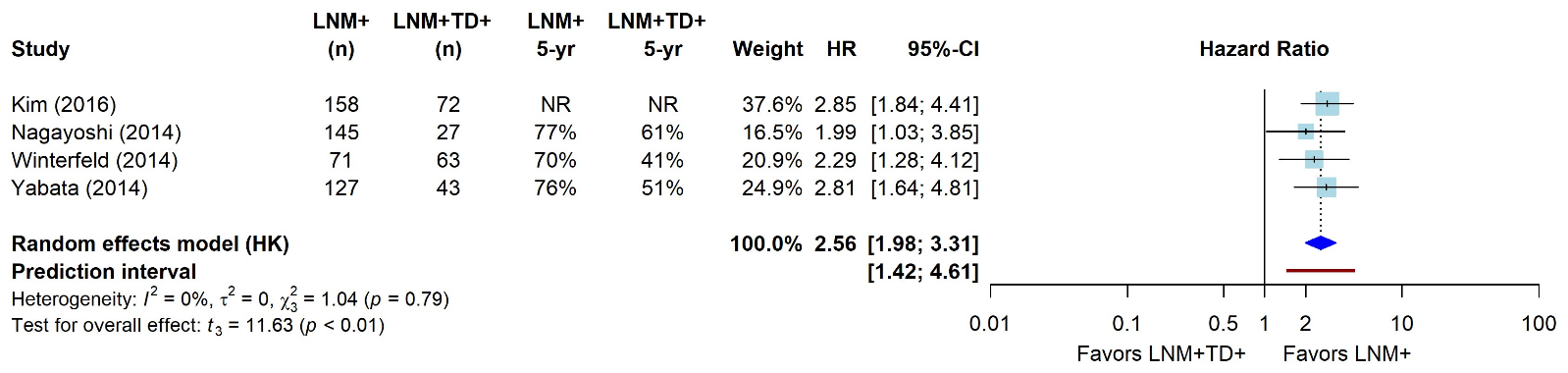

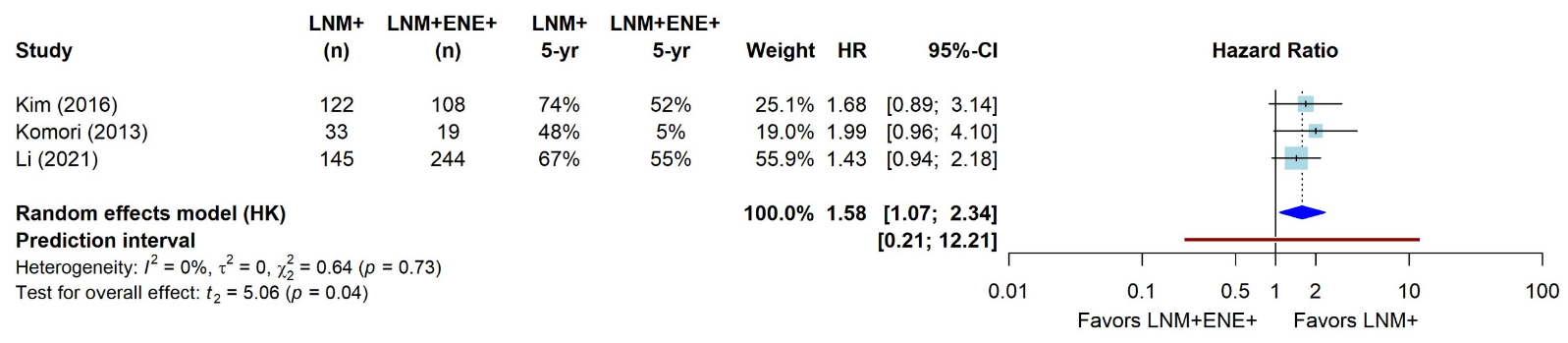


**Table S3**

Mixed effect estimates from the network meta-analyses for the different multivariable survival outcomes. Significant hazard ratios are coloured blue and not significant hazard ratios are coloured orange.

| Outcomes | Number of cohorts (patients) | Comparison | LNM+ | TD+ | LNM+ENE+ |
| --- | --- | --- | --- | --- | --- |
| Disease-free survival | LNM+: 13 (3231) |  |  |  |  |
|  | TD+: 0 (0) | TD+ | - | - | - |
|  | LNM+ENE+: 7 (1392) | LNM+ENE+ | 1.53 [1.29; 1.80] | - | - |
|  | LNM+TD+: 6 (466) | LNM+TD+ | 1.72 [1.41; 2.10] | - | 1.13 [0.87; 1.46] |
|  |  |  |  |  |  |
| Disease specific survival | LNM+: 4 (317) |  |  |  |  |
|  | TD+: 0 (0) | TD+ | - | - | - |
|  | LNM+ENE+: 2 (177) | LNM+ENE+ | 1.32 [0.94; 1.87] | - | - |
|  | LNM+TD+: 2 (96) | LNM+TD+ | 2.41 [1.63; 3.58] | - | 1.82 [1.08; 3.08] |
|  |  |  |  |  |  |
| Overall survival | LNM+: 7 (801) |  |  |  |  |
|  | TD+: 0 (0) | TD+ | - | - | - |
|  | LNM+ENE+: 3 (371) | LNM+ENE+ | 1.58 [1.16; 2.17] | - | - |
|  | LNM+TD+: 4 (205) | LNM+TD+ | 2.56 [1.96; 3.34] | - | 1.62 [1.07; 2.44] |
